# Colicins and T6SS-based competition systems enhance enterotoxigenic *E. coli* (ETEC) competitiveness

**DOI:** 10.1080/19490976.2023.2295891

**Published:** 2023-12-27

**Authors:** Jonas Kjellin, Danna Lee, Hans Steinsland, Rachel Dwane, Oda Barth Vedoy, Kurt Hanevik, Sanna Koskiniemi

**Affiliations:** a Department of Cell and Molecular Biology, Uppsala University, Uppsala, Sweden; b CISMAC, Centre for International Health, Department of Global Public Health and Primary Care, University of Bergen, Bergen, Norway; c Department of Biomedicine, University of Bergen, Bergen, Norway; d Department of Clinical Science, University of Bergen, Bergen, Norway; e National centre for Tropical Infectious Diseases, Department of Medicine, Haukeland University Hospital, Bergen, Norway

**Keywords:** ETEC, colicin, type VI secretion, competitive advantage, genome

## Abstract

Diarrheal diseases are still a significant problem for humankind, causing approximately half a million deaths annually. To cause diarrhea, enteric bacterial pathogens must first colonize the gut, which is a niche occupied by the normal bacterial microbiota. Therefore, the ability of pathogenic bacteria to inhibit the growth of other bacteria can facilitate the colonization process. Although enterotoxigenic *Escherichia coli* (ETEC) is one of the major causative agents of diarrheal diseases, little is known about the competition systems found in and used by ETEC and how they contribute to the ability of ETEC to colonize a host. Here, we collected a set of 94 fully assembled ETEC genomes by performing whole-genome sequencing and mining the NCBI RefSeq database. Using this set, we performed a comprehensive search for delivered bacterial toxins and investigated how these toxins contribute to ETEC competitiveness *in vitro*. We found that type VI secretion systems (T6SS) were widespread among ETEC (*n* = 47). In addition, several closely related ETEC strains were found to encode Colicin Ia and T6SS (*n* = 8). These toxins provide ETEC competitive advantages during *in vitro* competition against other *E. coli*, suggesting that the role of T6SS as well as colicins in ETEC biology has until now been underappreciated.

## Introduction

Enterotoxigenic *Escherichia coli* (ETEC) is a major cause of bacterial diarrhea, resulting in about 220 million diarrheal episodes annually.^1^ In 2016, ETEC was the eighth leading cause of diarrhea mortality among all age groups, accounting for around 50 000 deaths, 18 000 of these in children below 5 years of age.^2^ In order to cause disease, ETEC enters the human host via the fecal-oral route and colonizes the small intestine, where it induces acute watery diarrhea by secreting diarrhea-inducing enterotoxins. Human ETEC is defined as *E. coli* that can produce at least one of two protein enterotoxins: heat-labile toxin (LT) and heat-stable toxin (ST)^3^. Recent phylogenetic studies have suggested that ETEC has emerged from the *E. coli* population on several different occasions through the acquisition of enterotoxin genes. Today, several of the most successful ETEC families are found in endemic areas worldwide.^4–7^

To effectively colonize the gut, ETEC must successfully navigate the host’s mucosal defenses and anchor itself to the small intestinal cell wall. It is not clear how this anchoring occurs in all ETEC strains, but colonization factors (CFs) are known to play an important role in the strains encoding them^8^. The CFs are adhesive fimbrial, fibrillar or other surface proteins that attach to enterocytes in the proximal small intestine. About two thirds of ETEC strains express at least one of the more than 25 known CFs^9^. Recent studies suggest that the ability of ETEC to successfully colonize humans may vary depending on the volunteer and the strain used.^10–12^

For successful colonization, ETEC must also challenge the presence of the existing normal gut microbiota. Gut microbiota can inhibit enteric pathogen colonization and expansion, a property termed colonization resistance.^13^ This resistance is partly due to the spatial occupancy of the niche by the normal gut microbiota, limited nutrient availability, and modulation of immune defense. Another important component of colonization resistance is comprised of bacterial toxin delivery systems, which many gut bacteria harbor and use for competition with other bacteria (reviewed in.^14^ Thus, the ability to deliver antibacterial toxins may play a role in ETEC colonization.

These antibacterial toxins can be secreted, such as bacteriocins, or delivered through direct cell–cell contact via contact-dependent growth inhibition *(cdiBAI*) or the type VI secretion system (T6SS). Bacteriocins produced by *E. coli* are divided into two classes, depending on their size: colicins (>10 KDa), and microcins (<10 KDa) (reviewed in^15^). Both classes are either secreted into the extracellular milieu through general Sec-mediated protein secretion, or released upon lysis of the producing cells. Once in the supernatant, colicins, and microcins target closely related Gram-negative bacteria. Since their discovery in 1925, more than 20 different types of colicins have been identified.^15^ While the function of colicins has been extensively studied, their role and importance during host colonization is still poorly understood. Samuels et al. found, for example, that the ability to produce colicin did not increase the colonization ability of *E. coli* when tested in a mouse model,^16^ whereas Sassone-Corsi et al. found that secreting microcins greatly improved colonization ability.^17^

An alternative mechanism of antibacterial toxin delivery is the use of CDI or T6SSs to transfer toxins directly into neighboring bacteria.^18,19^ CDI systems are encoded from a three gene cluster, *cdiBAI*, where CdiB is the outer-membrane transporter that transports the large, toxin containing CdiA protein to the cell surface.^20^ Upon direct contact with a target bacterium expressing a cognate outer-membrane receptor, the C-terminal end of the CdiA protein (encoding the toxin) is cleaved off and delivered to the targeted cell inhibiting its growth.^21^ On the other hand, T6SS comprises a versatile cell-puncturing device, capable of injecting a cocktail of effectors into prokaryotic competitors as well as into eukaryotic host cells (reviewed in^22^). The apparatus is composed of 13 core components, and the system has been shown to provide a significant competitive advantage to bacteria during competition *in vitro*.^23^ The effectors are attached to either the VgrG (*tssI*) tip,^24^ the Hcp (*tssB*) tube^25^ or to the PAAR protein,^26^ depending on their size and protein domains. Effectors can be divided into two classes; i) cargo effectors, where the effector is attached to any one of the above mentioned T6S apparatus components through protein–protein interactions or ii) evolved effectors, where the effector domain is part of either VgrG^27^ or Hcp.^28^ Antibacterial effectors have diverse activities with periplasmic, for example peptidoglyconases,^23^ or cytoplasmic, for example DNase, targets.^29^ Thus, collected evidence suggests that the presence of an active T6SS improves the colonization ability of pathogens.^30,31^ T6SS systems have been found in many pathogenic *E. coli*, including isolates of extraintestinal pathogenic (ExPEC), avian pathogenic (APEC), and enteroaggregative (EAEC) *E. coli*.^32^ In EAEC, up to two different T6SS (type i1 and i3) can be found in a single strain,^33^ whereas up to three complete T6SS systems (types i1, i2, and i3) have been found in APEC strains.^34^ Having multiple T6S effectors has been shown to result in synergy between different effectors,^35^ but why some bacteria have multiple T6SS loci is not clear. In addition, very little is known about the prevalence and use of T6SS in other *E. coli* including ETEC.

Infant intestinal microbial composition is likely to contribute to ETEC colonization resistance, which ETEC need to overcome to cause infection. To improve our understanding of competition systems in ETEC and how they contribute to successful ETEC colonization, we investigated the presence of bacterial competition systems in ETEC. To this end, we collected a large set of high-quality ETEC assemblies by mining publicly available complete *E. coli* genome sequences and by sequencing additional ETEC strains. We analyzed the presence and conservation of potential bacterial toxins in these genomes. To investigate the contribution of the identified competition systems to ETEC competitiveness, we performed knockout mutagenesis and *in vitro* competition assays, using strains that we could acquire and which contained different effector arsenals or types of systems. In total four strains were included in the study. Two of these strains contained an intact type i1 locus, one in combination with a partial type i2 locus and one in combination with a colicin. The two remaining strains carried partial i1 loci, one where this was complemented with a partial type i2 locus, and one where no complementation existed. The latter was included as a control which should not show T6SS activity. Our results suggest that competition systems are abundant in ETEC genomes, and that the genes encoding the type i1 T6SS are in particularly highly represented. In addition, we show that these systems are under stabilizing selection and provide evidence that some of these systems contribute to the competitive ability of ETEC *in vitro*.

## Results

### ETEC identification and phylogeny

To identify antibacterial toxins in ETEC, we collected a set of 90 ETEC genome sequences by searching for the structural genes of the porcine (STp) and human (STh) variants of ST, as well as LT in all completely assembled *E. coli* genome sequences available in the NCBI RefSeq database (*n* = 2333, accessed October 4, 2022). In addition, we sequenced the genomes of four additional ETEC strains (TW10573, TW10828, TW14425, and TW10690), as described previously.^5,36^ High-quality nanopore genome sequences with > 100× coverage were obtained for all four sequenced strains, enabling the successful assembly of each chromosome and plasmid into single contigs (Table S1). Similarity was assessed for assemblies with same sequence type or similar repertoire of competition systems (see below) to control for redundancy in the data set (Table S11). Detailed information on all 94 ETEC strains, including the predicted toxin and CF profiles, can be found in Table S2.

**Figure 1.**
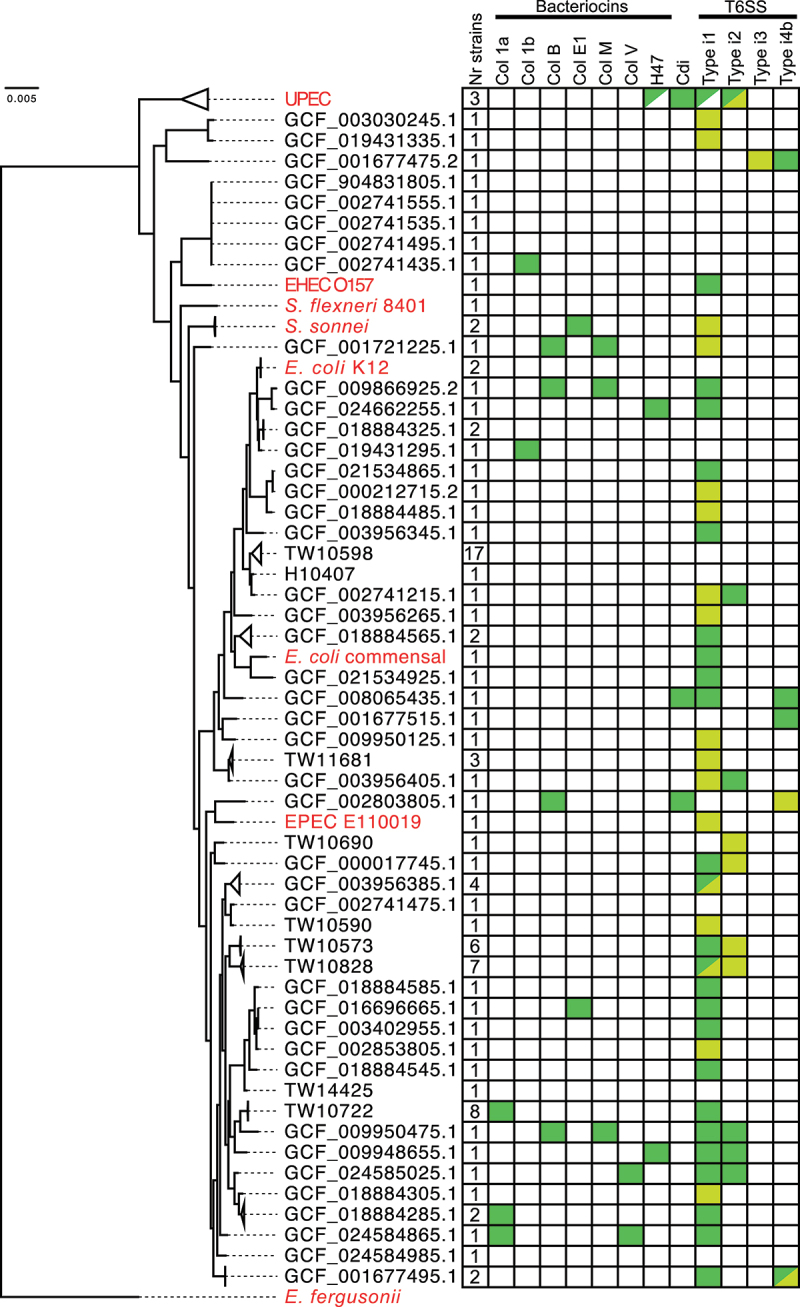
Phylogeny and competition factors of ETEC strains.

To assess the relatedness between these strains, we identified, concatenated, and aligned 100 single-copy genes found in all the ETEC strains and 11 non-ETEC *E. coli* and *Shigella* reference strains. Using this alignment, we generated a phylogenetic tree using *Escherichia fergusonii* as an outgroup (Figure 1, Fig. S1, Table S2). To determine the representativeness of the ETEC strains included in this study, we compared the sequence type and CF repertoire of the ETEC strains with previously reported lineages from von Mentzer et al.^7^ All 10 previously reported lineages were represented in our dataset by 50 ETEC strains. Some of these lineages are well represented, for example, L2 and L5 with nine and eight strains, respectively, whereas only one strain with the same sequence type and CF repertoire could be identified for L9 and L10, respectively (Table S2). Furthermore, we found 23 ETEC strains in which we could not identify any CFs, and 21 strains with a combination of sequence type and CF, which has not been attributed to any of the lineages reported by von Mentzer et al. (Table S2).

Relatedness and identified competition factors of 94 ETEC strains and 11 non-ETEC *E. coli* and *Shigella* strains using *E. fergusonii* as outgroup. All ETEC strains are named after respective assembly RefSeq accession number or TW name. Names of non-ETEC strains are in red. Some branches were collapsed based on the presence of bacteriocins and T6SS and named by a representative member of the group. Number of strains present in a collapsed branch is indicated. Green indicates the presence of a bacteriocin or T6SS. Half-filled box indicates that some but not all members of the group (collapsed branches) carry the bacteriocin or T6SS (Full tree in Fig. S1). Yellow indicates partial T6SS system.

### Lineage 5 ETEC strains encode Colicin Ia

To study the bacterial competition systems in ETEC, we first searched the genome of each strain for genes encoding bacteriocins. Using the sequences of all proteins annotated as bacteriocins in the UniProt database (3753 unique sequences, release 2022_04) as a reference, we identified different colicins or microcins in 21 of the 94 ETEC strains (Table S2). The majority of these strains, 17 out of 21, encode only one colicin or microcin, whereas the remaining four each have a combination of two different colicins, either the combination of Colicin B/M (three strains) or Colicin Ia/V (one strain). Colicin Ia, which was identified in 11 different strains, was the most prevalent bacteriocin (Table S2).

Eight of the 11 strains encoding Colicin Ia belonged to the ETEC Lineage 5 (Figure 1, Fig. S1). Among these eight strains, the Colicin Ia enocding gene sequences were identical (100% nucleotide conservation) and present on ~ 150 kbp plasmids that shared > 99.9% average nucleotide sequence identity. The assemblies of the eight Colicin Ia encoding ETEC strains are 91.7–99.9% similar to each other (Table S11) and were collected from human stool samples over a period of 15 years from five different countries (Table S3). We identified no strains belonging to lineage 5 lacking the Colicin Ia-encoding plasmid, suggesting that it is under selective pressure to be maintained in the ETEC genomes. In support of this, the plasmid encoding Colicin Ia was the only plasmid found in all eight lineage 5 strains, while the presence of other plasmids appeared to be more dynamic (Fig. S2A). We also compared all plasmids found in the nine-member lineage 2 strains in our dataset and found that plasmids appeared to be frequently gained and lost within this lineage (Fig. S2B). Another example of a plasmid conserved within an entire multi-member clade (five members) was identified (L7) (Table S2, Fig. S2C). Interestingly, this plasmid encodes both the enterotoxin and colonization factor for these strains, suggesting that it is also under strong stabilizing selection. The remaining three strains encoding Colicin Ia, belonged to two other clades in the ETEC phylogeny (Figure 1). The Colicin Ia-encoding plasmids of these strains differed from those found in the lineage 5 strains and showed low levels of synteny in the genes surrounding the *col-Ia* gene (encoding Colicin Ia) (Fig. S3). However, the *col-Ia* gene is highly similar in all strains it was identified and has > 99% sequence similarity to the genes found in lineage 5 strains (Table S4). None of the strains belonging to either of these two clades lacked *col-Ia*. Taken together, we found no evidence of an ETEC lineage losing the *col-Ia* gene once it had been acquired. This, together with the fact that the nucleotide sequence of *col-Ia* within each cluster is identical, suggests that the Colicin Ia-encoding gene is under strong stabilizing selection in these lineages.

### T6SS is a common contact dependent competition system for ETEC

Next, we analyzed competition systems that require direct cell-to-cell contact between cells, that is, the presence of *cdiBAI* or core genes required for a functional T6SS. T6SS is classified into different subtypes depending on the sequence and order of the core components. We found that 47 (50%) of the 94 ETEC strains encoded potentially functional T6SS, and seven of the strains encoded two systems (Figure 1, Table S2). The majority (43 of 47) of the strains with potentially functional T6SS systems had the type i1 locus, of which 38 had all core component genes intact in the type i1 locus and five had genes split between a type i1 locus and either a type i2 or a type i4 locus (Table S2). Two other types of intact T6SS were also identified: type i2 (five strains) and type i4b (four strains). One of the strains with intact type i4b system (GCF_001677475.2), also encodes an almost intact type i3 system (Table S2). Representative gene orders of identified T6SS types are found in Fig. S4A and an overview of the genomic localization of the different systems can be found in Fig. S4B. The strains encoding type i2 or type i4b T6SSs were not closely related, as indicated by their phylogeny (Figure 1). Type i4b systems were exclusively found on large > 100kbp plasmids in this data set. In contrast, strains encoding type i1 T6SS were grouped together in the phylogenetic analysis. For example, all strains belonging to lineage 5 harbored intact type i1 T6SS. Taken together, these results show that T6SS are widespread among ETEC strains, where sub-type i1 is in particular highly represented.

**Figure 2.**
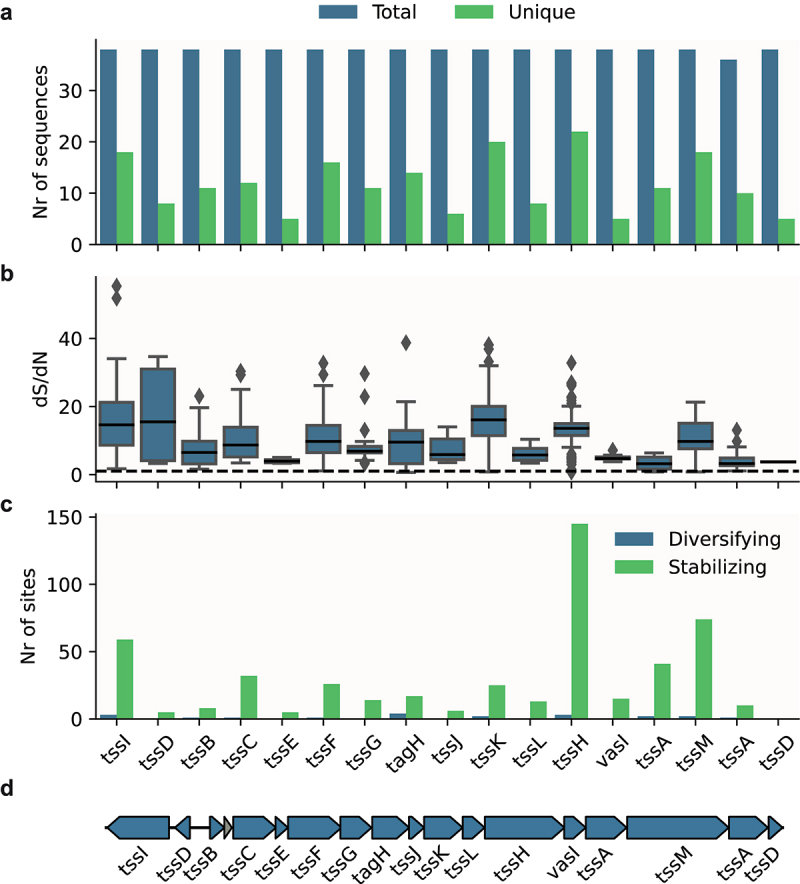
Conservation and selective pressure of type i1 T6SS components in ETEC strains with potentially active systems.

We also identified partial or fragmented systems in 22 of the 94 ETEC strains. As these strains lack core components of the T6SS, these systems are unlikely to be active or contribute to ETEC fitness. Fragmented genes, due to the accumulation of non-synonymous mutations, are a hallmark of reductive evolution due to purifying selection.^37^ As most mutations are deleterious in nature, genomic DNA under stabilizing selection is characterized as regions where synonymous mutations occur more frequently than non-synonymous mutations.^38,39^ Therefore, we investigated whether the intact type i1 systems (*n* = 38) were under purifying, stabilizing, or diversifying selection. We identified low sequence diversity among the T6SS core genes in these strains (Figure 2a), suggesting that these systems are under stabilizing selection. We also found evidence of stabilizing selection when we analyzed the dS/dN ratio for each gene with SNAP (Figure 2b, Table S5) and for every codon in each gene with FUBAR (Figure 2c, Table S6). A representative type i1 locus can be seen in Figure 2d. Taken together, these results suggest that T6SS is important for ETEC, as they are under selection pressure to be kept intact.

a) Nucleotide sequence complexity for each T6SS component in strains with potentially active systems. b) dS/dN values for the unique sequences for each T6SS component. Dashed line = 1 and dS/dN above 1 suggest stabilizing selection. c) Number of codons in the nucleotide sequence of each component with indication of stabilizing or diversifying selection. Gene order corresponds to the T6SS locus represented by TW10722 type i1 in **d**.

In contrast to T6SS, most ETEC strains do not appear to use contact-dependent growth inhibition (CDI) for competition. Full CDI systems (containing the *cdiBAI* gene cluster) were only detected in two of the ETEC strains, and these did not group phylogenetically (Figure 1, Table S2). This is lower than expected from all *E. coli* genomes, where recent analysis shows that CDI systems are found in 7% of genomes (Muir et al. in prep).

### ETEC strains encode an arsenal of rhs effectors

The conservation of T6SS prompted us to investigate whether ETEC strains share conserved effectors. T6SS effectors can be divided into specialized effectors, which are characterized by a Hcp, PAAR or VgrG domain followed by a C-terminal toxin extension, or cargo effectors which interacts non-covalently with the T6SS machinery. To identify putative specialized effectors, we analyzed all ETEC strains with potentially active systems for protein sequences with Hcp, VgrG, or Hcp domains. In order to identify putative cargo effectors, we searched for protein sequences with domains previously shown to be hallmarks of T6SS effectors, that is, MIX,^40^ FIX^41^ and RIX.^42^ Using this approach, we identified 279 putative specialized effectors but no cargo effectors. It is likely that these strains do encode cargo effectors that do not have the MIX, FIX or RIX domain. However, currently we do not have a reliable way to identify them bioinformatically.

**Figure 3.**
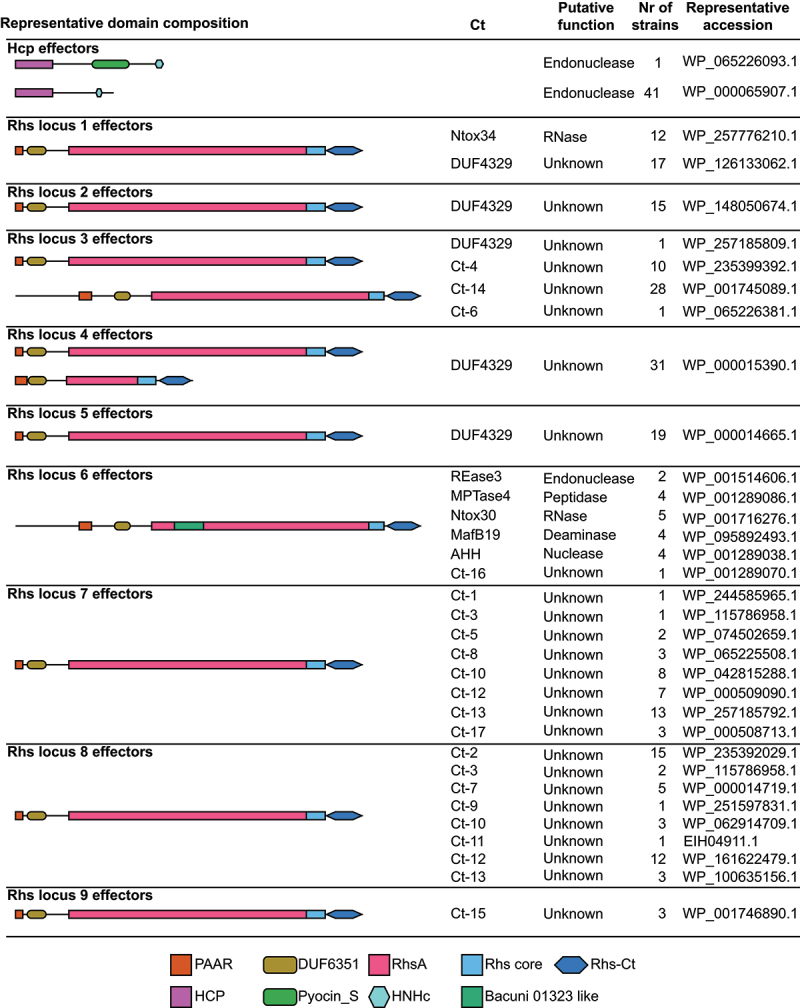
Representative domain composition of putative T6SS effectors identified in ETEC strains with potentially active T6SS (*N* = 47).

We identified two types of potential effectors with Hcp domains (Figure 3). The first, which also contains S-type pyocin and HNH nuclease domains, was only present in one of the ETEC strains. The other, with only Hcp and HNH nuclease domains, was present in more than 41 out of 47 ETEC strains with potentially active type i1 T6SS (Fig. S1, Table S2).

Putative function, how many strains it was identified in and representative accession is summarized for each type of effector. Rhs effectors are grouped based on their genomic location. Details of each Rhs locus is found in Fig. S5.

We identified 237 intact proteins with PAAR domains of which all were found to be rearrangement hotspot (Rhs) proteins, a known class of T6SS effectors.^29^ All of the investigated ETEC strains harbored three to nine different Rhs effectors (Fig. S1, Table S2). The Rhs proteins are modular, with a C-terminal toxin domain encapsulated in a YD-repeat cocoon.^43^ We further characterized the Rhs proteins by clustering them based on synteny and sequence similarity of the C-terminal toxin part. In total, we identified 9 different Rhs loci and 24 different toxins. A schematic overview of where these loci can be found on the *E. coli* chromosome is found in Fig. S4B. Of the 24 Rhs toxins, six have a predicted activity while the functions of the remaining 18 toxins are unknown (Figure 3). 37 Rhs effectors were found to be associated with type i1 T6SS locus (locus 7) and genes encoding VgrG were found upstream of the majority of Rhs effectors in loci 6, 7, 8 and 10 (Fig. S5). Rhs effectors in locus 7 are found adjacent to the type i1 T6SS except for in GCF_001677475.2 which lacks type i1 (Figure 3, Table S2). In general, strains belonging to the same clade in phylogenetic analysis also carry a similar arsenal of effectors. However, there are also examples where closely related strains have different Rhs toxins. One example was found when comparing the two closely related L3 strains, TW10828 and ETEC-2264 (GCF_002302335.1) (Fig. S1, Table S2). Both strains encode an Rhs toxin with a nuclease AHH domain in locus 6. However, in the same locus, ETEC-2264 contains an Rhs toxin with a nucleotide deaminase MafB19 domain that is lacking in TW10828 (Table S2).

### *ETEC strains expressing colicin can outcompete* E. coli *MG1655* in vitro

**Figure 4.**
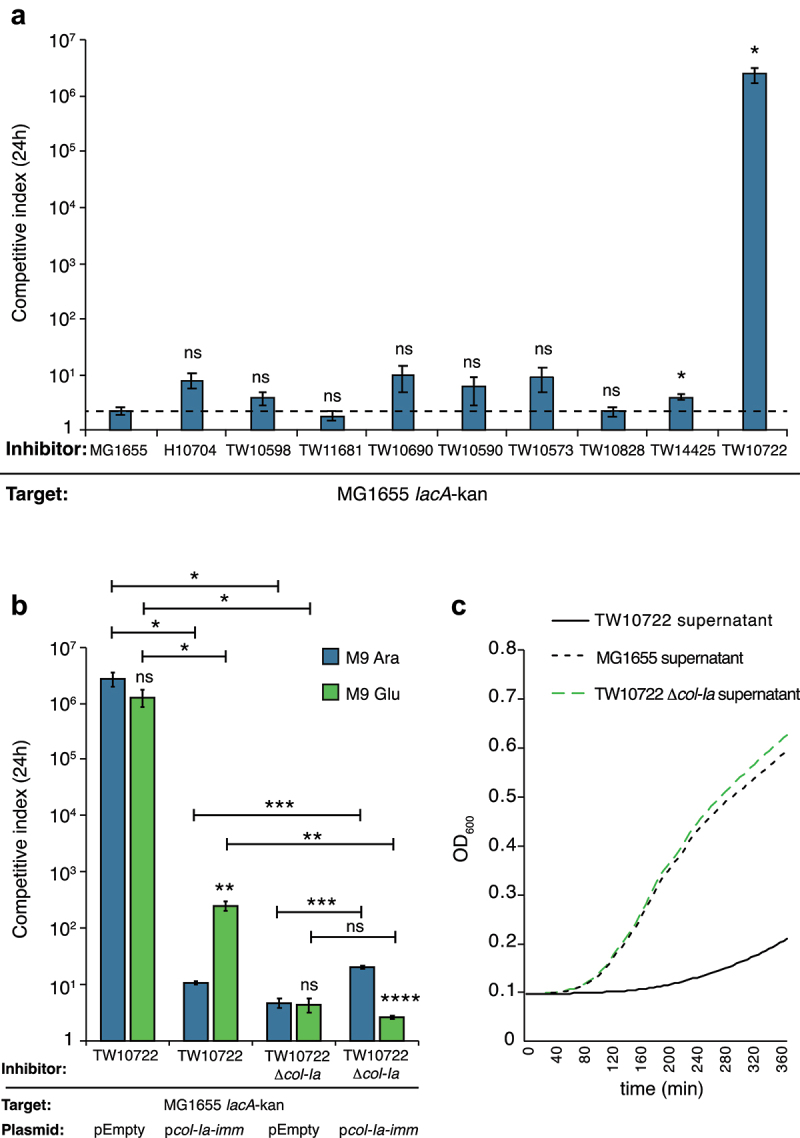
ETEC TW10722 uses Colicin Ia to outcompete MG1655 *in vitro*.

To determine whether any of the competition systems identified in the ETEC strains were active against other *E. coli*, we performed competition experiments with nine of the strains included in our dataset. We co-cultured them at a ratio of 1:1 with an *E. coli* MG1655 strain carrying a neutral chloramphenicol marker downstream of *lacA* (*lacA-cat*)^44^ on solid M9 minimal medium for 24 h. One ETEC strain, TW10722 of the L5 family, outcompeted *E. coli* MG1655 by five to six orders of magnitude on solid media (Figure 4a, Table S7). No significant competitive advantage was observed for any of the other ETEC strains, except TW14425 which displayed a 4-fold increase in competitive index over the MG1655 target strain. However, also the wild-type MG1655 displayed a 2-fold competitive advantage over the MG1655 target strain with *lacA*-kan, suggesting that this target strain has a small fitness disadvantage in this media. Several of the tested strains encoded T6SS, but only TW10722 encoded Colicin Ia, suggesting that this colicin could be responsible for the observed inhibition.

a-b) Competitive index of ETEC strains co-cultured at a ratio of 1:1 with *E. coli* MG1655 on M9-glycerol solid media for 24 h as such (A) or supplemented with 0.2% L-arabinose or glucose (B). Strains were separated by plating on different antibiotics. Error bars represent SEM of *N* = 6 biological replicates. a) ETEC co-cultured against *E. coli* MG1655, b) ETEC TW10722 wild-type or *col-Ia:cat* mutant co-cultured with MG1655 supplemented with empty vector or vector encoded Colicin Ia immunity under an arabinose inducible promoter. Statistical significance was determined using Student’s t-test for A and two-way Anova with Fisher’s LSD post hoc test for B. ns= not significant, *= *P* < 0.05, **= *P* < 0.01, ***= *P* < 0.001 and ****= P < 0.0001. Significance stars above the bars indicate significant difference compared to the MG1655 vs MG1655 control competition (A) or for that competition in the two different media (M9 Glu and M9 Ara) (B). Other relevant statistical analyses are indicated with lines connecting the two samples that were compared. All statistics can be found in Table S7. c) The growth of *E. coli* MG1655 in M9-glycerol supplemented with spent media from wildtype *E. coli* MG1655, TW10722, or TW10722 *col-Ia:cat* cultures. Growth is measured by OD_600_ measurements over a 6 h period.

To investigate whether Colicin Ia is responsible for the inhibition observed above, we i) deleted the Colicin Ia-encoding gene in TW10722, and ii) cloned the Colicin Ia immunity gene, *col-Ia-imm* under an arabinose-inducible promoter on pBAD33 and transformed it, along with an empty vector control, into MG1655. TW10722, lacking *col-Ia*, did not outcompete MG1655 (Figure 4b). At the same time, wild-type TW10722 did not outcompete MG1655 carrying pBAD33:*col-Ia-imm* when immunity expression was induced through growth on arabinose containing media (Figure 4b, Table S7). A small change in competitive index (~10-fold) was observed in the strain with the pBAD33:*col-Ia-imm* plasmid on arabinose, but this is likely due to a fitness cost from expressing *col-Ia-imm*, as a similar change could be seen when the same strain was competed against TW10722 lacking *col-Ia*, but not for the empty vector (Figure 4b, compare blue bars). On M9-glucose, the presence of the immunity plasmid still provided 3-logs of protection (Figure 4b), but this was significantly different from the protection observed on arabinose. The pBAD promoter is known to be leaky, and with a strong RBS some protein is likely to be produced also during repressive conditions. Thus, the partial protection observed on glucose is a likely indication of how little immunity is required for protection against the Colicin Ia producing strain. To further verify that the secreted colicins from TW10722 inhibited the growth of MG1655, a supernatant growth assay was performed. In essence, supernatants from centrifuged overnight cultures of MG1655, TW10722, or TW10722 *col-Ia:cat* were filter-sterilized and added to growth media (ratio 1:1 supernatant: LB). MG1655 was able to grow well in the supernatant from MG1655 and TW10722 *col-Ia:cat*, whereas no growth was observed in the TW10722 supernatant (Figure 4c, Table S7). Taken together, these results suggest that Colicin Ia is produced by TW10722 and that its presence in the supernatant inhibits the growth of MG1655.

### *T6SS is activated by bile and contribute to ETEC competitiveness* in vitro

**Figure 5.**
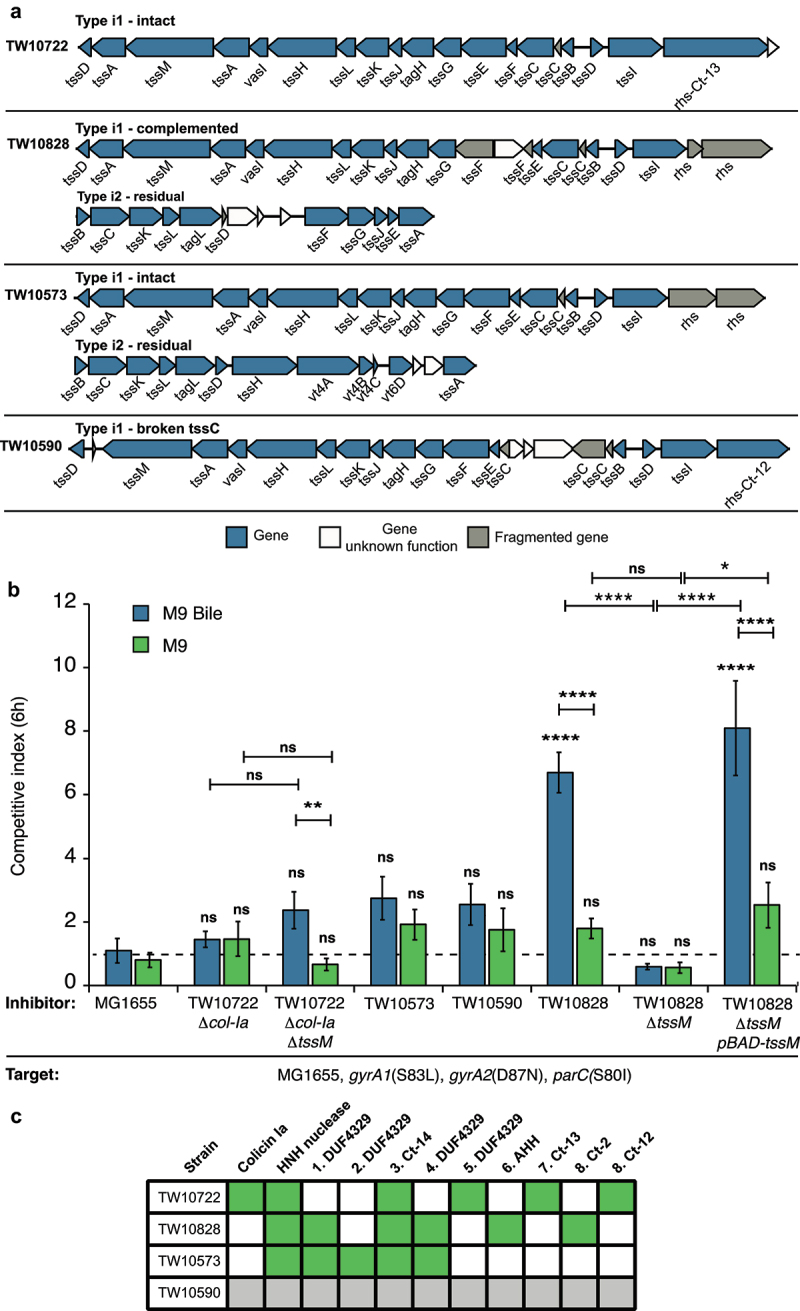
ETEC TW10828 uses T6SS to outcompete MG1655 *in vitro*.

Next, we investigated the activity of T6SS in three representative ETEC strains that we could acquire. We also included one ETEC strain with a broken T6SS (TW10573) as control. Two of these strains contained an intact type i1 system (TW10722 and TW10573) (found in the majority of ETEC strains with T6SS) and one a partial type i1 system, that was complemented with a partial type i2 system (TW10828). Also, TW10573 carried a partial type i2 system (Figure 5a). The effector arsenal among these three strains was also different (Figure 5c). Unfortunately, we were not able to acquire any of the strains with intact type i2 or type i4 systems and could therefore not assess the activity of these systems. To exclude the effect of colicins, we used a TW10722 strain lacking *col-Ia* for these competitions. To assess the effect of T6SS on competitive ability, we removed *tssM*, which encodes one of the core components of T6SS^45^ in TW10722 Δ*col-Ia* and TW10828. Unfortunately, TW10573 was not possible to transform, and we were unable to generate any deletion mutants in this strain. We competed all of these strains against MG1655 carrying neutral mutations providing resistance to ciprofloxacin (*gyrA1*(S83L), *gyrA2*(D87N), *parC*(S80I)).^46^ None of the strains showed a more than 2-fold competitive ability on M9 minimal medium supplemented with cas-amino acids and glycerol (Figure 5b, green bars). T6SS in other *Enterobacteriaceae* have been shown to be activated only upon specific environmental cues, such as bile.^30^ Therefore, we repeated the competition experiment on M9-media supplemented with cas-amino acids, glycerol, L-arabinose and 0.1% bile. We found that TW10828 could outcompete MG1655 6-fold under these conditions, whereas the no competitive ability could be observed for the mutant lacking *tssM* (Figure 5b, blue bars, Table S7). No inhibition could be observed for the other strains. This suggest T6SS was activated in TW10828 during growth on bile, and allowed TW10828 to inhibit the growth of MG1655. To ensure that this was due to inactivation of *tssM* and not because of other changes, we complemented the *tssM* mutant with a *tssM* expressed in trans from an arabinose inducible pBAD33 vector. Complementation of the *tssM* mutant with an arabinose inducible *tssM*, restored the competitive ability of TW10828 on bile (Figure 5b, Table S7), further supporting that T6SS is activated in TW10828 during growth on bile. In summary, our results show T6SS is active in ETEC TW10828 and that it contributes to the competitive ability of this strain *in vitro*.

a) Illustrations of the T6SS loci found in the different ETEC strains available for competitions (TW10722, TW10828, TW10573, and TW10590). b) Competitions with ETEC with or without *tssM* against *E. coli* MG1655 on solid M9-glycerol media supplemented with 0.1% bile salts and L-arabinose for 6 h. Strains were mixed 10:1 and competitive index was calculated as the change in ratio between 0 h and 6 h. Error bars represent SEM, *n* = 6. Statistical significance was determined using two-way ANOVA with a Fisher LSD post hoc test. ns= not significant, *= *P* < 0.05, **= *P* < 0.01, ***= *P* < 0.001 and ****= P < 0.0001. Significance stars above the bars indicate significant difference compared to the MG1655 vs MG1655 control competition in that media. Other relevant statistical analyses are indicated with lines connecting the two samples compared to each other. All statistics can be found in Table S7. c) T6SS effectors encoded by the ETEC strains used in competitions in **B**.

## Discussion

Some ETEC strains are more efficient in causing disease than others.^47^ Recent human clinical trials with ETEC infections suggest that some of this variation may be a result of differences in the colonization efficacy.^10–12^ However, the underlying reason why some ETEC are better colonizers than others is still unclear. Here, we provide evidence that the competitive ability of ETEC strains varies and that the acquisition of competitive systems is beneficial for the bacteria. This suggests that the competitive ability of ETEC could affect its colonization ability. On the other hand, ETEC shares a growth niche with other *Enterobacteriaceae* when found in the environment,^48^ and inter-bacterial competition abilities could therefore be important for survival outside the host. Hence, the presence of competition systems could represent ETEC’s ability to inhibit the growth of non-kin bacteria both outside and within the host.

We identified a set of closely related strains isolated from different geographical regions over several decades that all contain the same plasmid encoding Colicin Ia. The genomes of these ETEC isolates were 91.7–99.9% similar (nucleotide identity multiplied by alignment coverage), suggesting that the acquisition of Colicin Ia might have allowed the spread and persistence of a particular ETEC worldwide. The lack of evidence for ETEC loss of colicin suggests a strong positive selection for the gene but could also reflect the difficulty of losing a selfish genetic element.^49^ As Colicin Ia allows ETEC to outcompete other *E. coli*, it is possible that the presence of toxins produced by neighboring bacteria will kill any bacteria losing the system (in particular those losing immunity). Systems where the toxin can be delivered rather than only produced internally have been shown to have strong stabilizing effects on plasmids.^50^ Therefore, it is also possible that Colicin Ia is under positive selection not because it provides ETEC with a positive fitness gain but because it is challenging to lose once it has been acquired.

The ability to produce colicins is expected in the *Enterobacteriaceae* family, and studies have revealed that approximately 30% of *E. coli* strains produce at least one type of colicin.^51^ In this study, we identified colicins in 23% of ETEC genomes (Table S2), suggesting that colicins may be less common in ETEC than in *E. coli* in general. We also found no evidence for the positive selection of other colicins in our dataset. Colicin Ia is found in approximately 10% of *E. coli* strains of different origins, and at similar frequencies across *E. coli* groups in human strains.^52^ In our selection of ETEC, Colicin Ia was present in 12% of the strains, indicating a similar frequency as observed for *E. coli* in general. Colicin Ia is a 70 KDa protein atypical in that it is secreted to the extracellular milieu rather than being released by cell lysis,^15^ which could explain why this colicin is so frequent. The genes encoding Colicin Ia are often co-located with those encoding microcin M on a large conjugative plasmid ranging from 80 to 150 kbp in size.^52,53^ In our study, we also found *col-Ia* on a large conjugative plasmid that did not contain genes encoding microcin M. This plasmid also encodes the *pemIK* toxin-antitoxin system, which could potentially provide additional selection for the plasmid through post-segregational distortion upon plasmid loss.^54^

Half of the ETEC strains identified harbored T6SS, which is higher than that found in other classes of *E. coli*; e.g., only 14% of APEC (Avian Pathogenic *E. coli*) strains contain T6SS.^55^ Recent evidence suggests that T6SS is important for *Citrobacter rodentium* colonization of the gut, and that commensal *E. coli* can utilize their T6SS to prevent *Citrobacter* colonization.^31^ Therefore, the presence and positive selection of T6SS core genes suggests that some ETEC may also utilize their T6SS to compete with normal flora to establish infection in the host gut. This hypothesis is supported by the observation of antibacterial activity of T6SS type i1 found in at least one of the tested ETEC strains. In addition, bile salts were required to activate the system, suggesting a role for T6SS in host colonization. This is further supported by the fact that the presence of the T6SS type i1 system in various *E. coli* strains has been linked to increased virulence.^55,56^

The arsenal of the T6SS effector varied greatly among the different ETEC strains. Even phylogenetically closely related strains of ETEC showed the accumulation of new effectors, suggesting that effectors are frequently transferred by horizontal gene transfer between strains. The most prevalent effectors were Rhs toxins, which were found in all strains with complete T6SS. Rhs effectors are modular toxins consisting of an N-terminal delivery part and a C-terminal toxin, separated by a conserved Rhs core region. This modular structure allows the C-terminal toxin to be exchanged through homologous recombination.^57^ In this study, we find nine different loci where Rhs effectors are located and examples of closely related ETEC that encodes different repertoires of Rhs effectors. This suggests that the Rhs effector arsenal in ETEC is highly dynamic and might provide the bacteria with the opportunity to rapidly adapt their competitive ability. Rhs toxins have been shown to be important for virulence in *Salmonella*^58^ and *Pseudomonas*,^59^ and contribute to bacterial competition in a range of *Enterobacteriaceae*.^29,57,60^ However, not all Rhs effectors are antibacterials. Instead, some Rhs effectors mediate their effects on host cells,^59^ suggesting that the presence of T6SS and the associated Rhs effectors could also impact ETEC’s interaction with host cells. How the Rhs effector arsenal contributes to ETEC pathogenicity lies outside the scope of this work and will require future analyses.

In conclusion, our work suggests that competition systems are readily found in ETEC strains and that the systems we found are under stabilizing selection. Whereas most systems are found at similar prevalence in ETEC genomes as in other *E. coli* (e.g. colicins), the type i1 T6SS seems to be over-represented in ETEC strains. Why this is and if this T6SS has a function in ETEC pathogenicity, will be interesting to find out in future work. However, as no comprehensive analysis of T6SS prevalence has been carried out for *E. coli*, it is difficult to know-how specific this over-representation is to ETEC. Perhaps there are other pathogenic *E. coli* which also show similar prevalence of T6SS type i1. Another limitation of our study and to ETEC research in general, is the availability of ETEC strains to test in the lab. Bioinformatic analyses can only say so much about the function and activity of genes and in order to fully evaluate functions strains must be tested in a laboratory setting. With more ETEC strains available, the findings here could be stronger and help explain differences in T6SS activity observed between ETEC strains.

## Materials and methods

### Strains and growth conditions

The *E. coli* strains used in the present study are listed in Table S8. The eight ETEC strains used in competitions were previously isolated from young children during a prospective cohort study on the etiology of childhood diarrhea in Bissau, Guinea-Bissau in 1996–1998,^61,62^ and are representative of ETEC lineages that are commonly associated with childhood diarrhea.^5^ All strains were grown at 37°C in Lysogeny Broth (LB) with shaking at 200 rpm, or on LB plates supplemented with 1.5% agar, unless specified otherwise. For the competition assays solid M9 minimal medium (33.7 mM Na_2_HPO_4_, 22 mM KH_2_PO_4_, 8.55 mM NaCl, 9.35 mM NH_4_Cl, 2 mM MgSO_4_, 0.1 mM CaCl_2_, 1.5% agar) was supplemented with 0.2% casamino acids and a carbon source of 1% glucose, glycerol, or arabinose as specified for each experiment. Antibiotics were used when appropriate at the following concentrations chloramphenicol (CAM) 12.5 mg/L, kanamycin (KAN), 50 mg/L, cefotaxime (CEF) 10 mg/L.

### Strain knockout constructions

To evaluate the effects of Colicin Ia and T6SS on the competitive abilities of ETEC, we knocked out these genes in strains TW10722 (GCF_018884385.1) and TW10828 (GCA_032368225.1). The TW10722 *col-Ia* (EXA02_RS26115) /*tssM* (EXA02_RS06400) knockout and TW10828 *tssM* (QMY51_01415) knockout were constructed by lambda red recombineering. In short, resistance markers were amplified from pKD3/pKD4 using primer pairs SK2037 and SK2038/SK2041 and SK2042 (Table S9) for *col-Ia/tssM* inactivation, respectively, in TW10722, and with 2529 and 2531 for *tssM* inactivation in TW10828. The PCR products were electroporated into TW10722 or TW10828 cells expressing lambda proteins, as previously described,^63,64^ but with a modified pSIM5 vector where a CTX-M-15 gene replaces the *cat* gene. Transformants were plated on relevant antibiotics, and resistant colonies were verified as knockouts by PCR and Sanger sequencing across the resistance markers.

To protect MG1655 from Colicin Ia, the Colicin Ia immunity gene (EXA02_RS26110) was amplified from wild-type TW10722 using primers SK2053 and SK2054 (Table S9) and cloned into pBAD33^65^ between the SmaI and SalI restriction sites. To complement the *tssM* knock-out, *tssM* (QMY51_01415) was amplified from wild-type TW10828 using oligos SK2727 and SK2728 (Table S9) and cloned into pBAD33 using XbaI and HindIII. Successful clones were verified by sequencing using oligos SK387 and SK388.

### Solid competition assay

In these assays, we tested the extent to which different ETEC strains (inhibitors) could compete with *E. coli* K-12, sub-strain MG1655 (target). MG1655 does not encode known competition systems and was used as target. Insertion of a neutral kanamycin or chloramphenicol marker downstream of *lacA* (*lacA-kan or lacA-cat*) enables selection and enumeration of targets.^44^ The ETEC inhibitor and MG1655 target strains were cultured independently overnight in LB, centrifuged, and washed once in sterile filtered Phosphate Buffered Saline (SF-PBS) before mixing at a ratio of 10:1 (Figures 4a,b and 5b) in PBS. For competition, 20 μl of these mixtures were spotted on solid M9 minimal media and incubated at 37°C for 24 h before resuspending the cells in PBS. For Colicin Ia immunity complementation competitions, the competition mix was spotted on solid M9 minimal media supplemented with either 1% glucose or 1% arabinose instead, and for T6SS competitions, on solid M9 minimal media supplemented with 1% arabinose, 1% glycerol, in the presence or absence of 0.1% bile extract porcine (Sigma) was used. At both 0 h and 6 h, colony-forming units per milliliter (CFU/ml) of the targets and inhibitors were scored on LA plates (for inhibitors) and plates with chloramphenicol or kanamycin (for targets) depending on the competition. Competitive indices were calculated as the change in the ratio of inhibitor to target cell concentration at 0 h as compared to either 6 h or 24 h. For details on which target strain, initial ratio of inhibitors and targets was used for each competition or how competitive indices were calculated please consult Table S7.

### Supernatant growth assay

To investigate the presence of secreted Colicin Ia in the supernatant, supernatants from MG1655, TW10722, and TW10722 Δ*col-Ia* cultures grown overnight were collected by pelleting the cells and filtering the supernatant. Overnight cultures of the MG1655 target strain were sub-cultured 1/200 in sterile filtered supernatant diluted 1× with fresh LB in a microtiter plate. The cells were grown at 37°C with shaking at 150 rpm for 6 h and OD_600_ measurements were made every 5 min over a 6 h period in an infinite M200 PRO microplate reader (Tecan Trading AG, Männedorf, Switzerland). Growth was analyzed as the increase in OD_600_ values over time.

## Whole genome sequencing of ETEC strains

Genomic DNA was isolated using the Genomic-tip 100/G method (Qiagen, Hilden, Germany) from the strains cultured overnight in LB broth. DNA concentration was measured using a Qubit 2.0 with dsDNA BR Kit (Invitrogen, Waltham, MA), and purity was determined by NanoDrop 1000 measurements. Barcoding and library preparation were performed using the Native Barcoding Kit 24 V14, followed by sequencing on an R10.4 flow cell in a MinION Mk1c device (Oxford Nanopore, Oxford, UK). Base calling and adapter trimming were performed with Guppy version 6.3.8+d9e0f64 (Oxford Nanopore) using the super-accuracy configuration (dna_r10.4.1_e8.2_260bps_sup). The resulting reads were then assembled using Flye v. 2.9.2-b1786^66^ and inspected using Bandage.^67^ Long-read polishing was performed using Medaka v. 1.0.3 (https://github.com/nanoporetech/medaka). For two strains, TW10573 and TW10690, paired-end Illumina libraries with 300 bp insert sizes were prepared using the NEBNext kit (New England Biolabs, Ipswich, MA), and 100 bp read-length sequencing was performed on an Illumina HiSeq 2000. The quality of these reads was inspected using FastQC and mapped to the long-read assemblies using BWA-MEM, version 0.7.17-r1188, followed by short-read polishing using Polypolish.^68^ All four genomes were then annotated with Prokka 1.14.6^69^ before being submitted as complete genomes to NCBI.

### ETEC collection and phylogeny

All complete *E. coli* genomes in NCBI RefSeq database (October 4, 2022) were downloaded by using the NCBI Datasets tool (github.com/ncbi/datasets). The ETEC colonization factor (CF) and enterotoxin sequences were collected from the NCBI protein database or UniProt (Table S10). The assemblies were then characterized based on the presence of LT and ST toxins, as well as CFs by using BLASTp^70^ with a 90% identity and coverage cutoff. All strains with LT and/or ST toxins were considered ETEC strains (Table S2). To facilitate comparisons with previous studies, 11 non-ETEC *E. coli* and *Shigella* strains used in the von Mentzer study^7^ were included as references.

Gene selection, alignment of core genes, and choice of outgroup organism for use in evaluating the relatedness between different strains were performed using autoMLST (-wf 1 -mo 0 -cat).^71^ Subsequent phylogenetic analysis, based on the maximum likelihood method, was performed with 1000 bootstrap replicates using IQ-TREE^72^ with a generalized time-reversible (GTR) substitution model. The resulting phylogenetic tree was drawn using figtree (https://github.com/rambaut/figtree). PubMLST multilocus sequence typing was performed *in silico* for all strains included in the tree, using mlst (https://github.com/tseemann/mlst).^73^ Average nucleotide identity and alignment coverage were analyzed for assemblies with the same sequence type and similar repertoire of competition systems (see below) with pyani v. 0.2.12^74^ (Table S11).

### Characterization of ETEC competition systems

All genomes included in the phylogeny were annotated with Prokka.^69^ Potential CDI genes were retrieved from Prokka annotations and evaluated manually. T6SS genes were identified by BLASTp^70^ using the SecReT6 database of experimentally validated T6SS components as reference.^75^ Coding sequences of type i1 T6SS components were codon-aligned with MACSE^76^ and dS/dN analyses were performed using SNAP^77^ and FUBAR.^78^ T6SS effectors were predicted in all strains with potentially functional systems by search all predicted proteins for Hcp, VgrG, PAAR, MIX, and FIX domains using rpsblast.^70^ We also searched for the presence of RIX domains by first performing five iterations of PSI-BLAST with the 55 N-terminal residues of Tme1 (WP_015297525.1) from *V. parahaemolyticus* BB22OP as described by.^42,42^ The result was aligned with MAFFT and used to construct a HMM profile of RIX with hmmbuild. The profile was validated against known RIX containing proteins and then used to search for RIX domain containing proteins in our dataset with hmmscan.^79^ Protein sequences with at least one domain hit and at least 80 amino acids following the domain were considered as putative effectors. Rearrangement hot spot (Rhs) effectors were further characterized by dividing them into two parts, delivery and toxin, by determining the location of the Rhs core domain with rpsblast. The toxin part of each Rhs were then clustered based on domain composition or, if no conserved domain could be identified, by sequence similarity with MMSeqs easy cluster.^80^

To identify and characterize bacteriocin systems, the complete set of *Escherichia* (taxid 561) bacteriocins was downloaded from UniProt (release 2022_04) and used to construct a BLAST protein database. The colicin repertoire for each strain was then determined by using BLASTp with a 90% coverage and 90% identity cut off. Accession number and sequence of all identified bacteriocins and T6SS effectors are found in Table S12. The average nucleotide identity of *col-Ia* was calculated using the OrthoANIu algorithm.^81^ Shared synteny mapping between plasmids between plasmids carrying Colicin Ia encoding genes was performed using Satsuma2 (https://github.com/bioinfologics/satsuma2). Comparison of all plasmids within lineages 2, 5 and 7 was performed using pyani v. 0.2.12.^74^ The genomic loci of Rhs effectors and *col-Ia genes* were extracted from annotations and plotted with DNA Features Viewer.^82^

## Supplementary Material

Table_S11.xlsx

Table S7 revised.xlsx

Table_S12.xlsx

Table_S2_revised_JK.xlsx

Table S6 revised.xlsx

Table S5 revised.xlsx

Supplemental_material_revised 231201 clean.docx

## Data Availability

All raw data for the manuscript are freely available either as supplementary excel files (Tables S5–8) or at the NCBI Reference Sequence Database (GCF_032367395.1, GCF_032365155.1, GCF_032366635.1, GCF_032368225.1).

## References

[1] Khalil I, Walker R, Porter CK, Muhib F, Chilengi R, Cravioto A, Guerrant R, Svennerholm AM, Qadri F, Baqar S, et al. Enterotoxigenic *Escherichia coli* (ETEC) vaccines: priority activities to enable product development, licensure, and global access. Vaccine. 2021;39(31):4266–19. doi:10.1016/j.vaccine.2021.04.018. 33965254 PMC8273896

[2] Collaborators, G. B. D. Diarrhoeal Disease. Estimates of the global, regional, and national morbidity, mortality, and aetiologies of diarrhoea in 195 countries: a systematic analysis for the global burden of disease study 2016. Lancet Infect Dis. 2018;18(11):1211–1228. doi:10.1016/S1473-3099(18)30362-1. 30243583 PMC6202444

[3] Zhang Y, Tan P, Zhao Y, Ma X. Enterotoxigenic *Escherichia coli*: intestinal pathogenesis mechanisms and colonization resistance by gut microbiota. Gut Microbes. 2022;14(1):2055943. doi:10.1080/19490976.2022.2055943. 35358002 PMC8973357

[4] Escobar-Paramo P, Clermont O, Blanc-Potard AB, Bui H, Le Bouguenec C, Denamur E. A specific genetic background is required for acquisition and expression of virulence factors in *Escherichia coli*. Mol Biol Evol. 2004;21(6):1085–1094. doi:10.1093/molbev/msh118. 15014151

[5] Steinsland H, Lacher DW, Sommerfelt H, Whittam TS. Ancestral lineages of human enterotoxigenic *Escherichia coli*. J Clin Microbiol. 2010;48(8):2916–2924. doi:10.1128/JCM.02432-09. 20534806 PMC2916599

[6] Turner SM, Chaudhuri RR, Jiang ZD, DuPont H, Gyles C, Penn CW, Pallen MJ, Henderson IR. Phylogenetic comparisons reveal multiple acquisitions of the toxin genes by enterotoxigenic *Escherichia coli* strains of different evolutionary lineages. J Clin Microbiol. 2006;44(12):4528–4536. doi:10.1128/JCM.01474-06. 17050815 PMC1698409

[7] von Mentzer A, Connor TR, Wieler LH, Semmler T, Iguchi A, Thomson NR, Rasko DA, Joffre E, Corander J, Pickard D, et al. Identification of enterotoxigenic *Escherichia coli* (ETEC) clades with long-term global distribution. Nat Genet. 2014;46(12):1321–1326. doi:10.1038/ng.3145. 25383970

[8] Madhavan TP, Sakellaris H. Colonization factors of enterotoxigenic *Escherichia coli*. Adv Appl Microbiol. 2015;90:155–197. doi:10.1016/bs.aambs.2014.09.003. 25596032

[9] Vidal RM, Muhsen K, Tennant SM, Svennerholm AM, Sow SO, Sur D, Zaidi AKM, Faruque ASG, Saha D, Adegbola R, et al. Colonization factors among enterotoxigenic *Escherichia coli* isolates from children with moderate-to-severe diarrhea and from matched controls in the global enteric multicenter study (GEMS. PLoS Negl Trop Dis. 2019;13(1):e0007037. doi:10.1371/journal.pntd.0007037. 30608930 PMC6343939

[10] Pop M, Paulson JN, Chakraborty S, Astrovskaya I, Lindsay BR, Li S, Bravo HC, Harro C, Parkhill J, Walker AW, et al. Individual-specific changes in the human gut microbiota after challenge with enterotoxigenic *Escherichia coli* and subsequent ciprofloxacin treatment. Bmc Genom. 2016;17:440. doi:10.1186/s12864-016-2777-0. PMC4898365 27277524

[11] Vedoy OB, Hanevik K, Sakkestad ST, Sommerfelt H, Steinsland H. Proliferation of enterotoxigenic *Escherichia coli* strain TW11681 in stools of experimentally infected human volunteers. Gut Pathog. 2018;10:46. doi:10.1186/s13099-018-0273-6. 30349586 PMC6192177

[12] Vedoy OB, Steinsland H, Sakkestad ST, Sommerfelt H, Hanevik K. Strong association between diarrhea and concentration of enterotoxigenic *Escherichia coli* strain TW10722 in stools of experimentally infected volunteers. Pathogens. 2023;12(2). doi:10.3390/pathogens12020283. PMC9960819 36839555

[13] Lawley TD, Walker AW. Intestinal colonization resistance. Immunology. 2013;138(1):1–11. doi:10.1111/j.1365-2567.2012.03616.x. 23240815 PMC3533696

[14] Boopathi S, Liu D, Jia AQ. Molecular trafficking between bacteria determines the shape of gut microbial community. Gut Microbes. 2021;13(1):1959841. doi:10.1080/19490976.2021.1959841. 34455923 PMC8432619

[15] Cascales E, Buchanan SK, Duche D, Kleanthous C, Lloubes R, Postle K, Riley M, Slatin S, Cavard D. Colicin biology. Microbiol Mol Biol Rev. 2007;71(1):158–229. doi:10.1128/MMBR.00036-06. 17347522 PMC1847374

[16] Samuels AN, Roggiani M, Smith KA, Zhu J, Goulian M, Kohli RM. Deciphering the role of colicins during colonization of the mammalian gut by commensal *E. coli*. Microorganisms. 2020;8(5). doi:10.3390/microorganisms8050664. PMC7284606 32370119

[17] Sassone-Corsi M, Nuccio SP, Liu H, Hernandez D, Vu CT, Takahashi AA, Edwards RA, Raffatellu M. Microcins mediate competition among *Enterobacteriaceae* in the inflamed gut. Nature. 2016;540(7632):280–283. doi:10.1038/nature20557. 27798599 PMC5145735

[18] Jurenas D, Journet L. Activity, delivery, and diversity of type VI secretion effectors. Mol Microbiol. 2021;115(3):383–394. doi:10.1111/mmi.14648. 33217073

[19] Ruhe ZC, Low DA, Hayes CS. Polymorphic toxins and their immunity proteins: diversity, evolution, and mechanisms of delivery. Annu Rev Microbiol. 2020;74:497–520. doi:10.1146/annurev-micro-020518-115638. 32680451 PMC8019152

[20] Aoki SK, Pamma R, Hernday AD, Bickham JE, Braaten BA, Low DA. Contact-dependent inhibition of growth in *Escherichia coli*. Science. 2005;309(5738):1245–1248. doi:10.1126/science.1115109. 16109881

[21] Ruhe ZC, Subramanian P, Song K, Nguyen JY, Stevens TA, Low DA, Jensen GJ, Hayes CS. Programmed secretion arrest and receptor-triggered toxin export during antibacterial contact-dependent growth inhibition. Cell. 2018;175(4):921–933 e14. doi:10.1016/j.cell.2018.10.033. 30388452 PMC6333426

[22] Coulthurst S. The type VI secretion system: a versatile bacterial weapon. Microbiology. 2019;165(5):503–515. doi:10.1099/mic.0.000789. 30893029

[23] Hood RD, Singh P, Hsu F, Guvener T, Carl MA, Trinidad RR, Silverman JM, Ohlson BB, Hicks KG, Plemel RL, et al. A type VI secretion system of *Pseudomonas aeruginosa* targets a toxin to bacteria. Cell Host Microbe. 2010;7(1):25–37. doi:10.1016/j.chom.2009.12.007. 20114026 PMC2831478

[24] Unterweger D, Kostiuk B, Otjengerdes R, Wilton A, Diaz-Satizabal L, Pukatzki S. Chimeric adaptor proteins translocate diverse type VI secretion system effectors in *Vibrio cholerae*. EMBO J. 2015;34(16):2198–2210. doi:10.15252/embj.201591163. 26194724 PMC4557670

[25] Silverman JM, Agnello DM, Zheng H, Andrews BT, Li M, Catalano CE, Gonen T, Mougous JD. Haemolysin coregulated protein is an exported receptor and chaperone of type VI secretion substrates. Mol Cell. 2013;51(5):584–593. doi:10.1016/j.molcel.2013.07.025. 23954347 PMC3844553

[26] Shneider MM, Buth SA, Ho BT, Basler M, Mekalanos JJ, Leiman PG. PAAR-repeat proteins sharpen and diversify the type VI secretion system spike. Nature. 2013;500(7462):350–353. doi:10.1038/nature12453. 23925114 PMC3792578

[27] Pukatzki S, Ma AT, Revel AT, Sturtevant D, Mekalanos JJ. Type VI secretion system translocates a phage tail spike-like protein into target cells where it cross-links actin. Proc Natl Acad Sci U S A. 2007;104(39):15508–15513. doi:10.1073/pnas.0706532104. 17873062 PMC2000545

[28] Blondel CJ, Jimenez JC, Contreras I, Santiviago CA. Comparative genomic analysis uncovers 3 novel loci encoding type six secretion systems differentially distributed in *Salmonella* serotypes. Bmc Genom. 2009;10:354. doi:10.1186/1471-2164-10-354. PMC2907695 19653904

[29] Koskiniemi S, Lamoureux JG, Nikolakakis KC, t’Kint de Roodenbeke C, Kaplan MD, Low DA, Hayes CS. Rhs proteins from diverse bacteria mediate intercellular competition. Proc Natl Acad Sci USA. 2013;110(17):7032–7037. doi:10.1073/pnas.1300627110. 23572593 PMC3637788

[30] Sana TG, Flaugnatti N, Lugo KA, Lam LH, Jacobson A, Baylot V, Durand E, Journet L, Cascales E, Monack DM. *Salmonella typhimurium* utilizes a T6SS-mediated antibacterial weapon to establish in the host gut. Proc Natl Acad Sci U S A. 2016;113(34):E5044–51. doi:10.1073/pnas.1608858113. 27503894 PMC5003274

[31] Serapio-Palacios A, Woodward SE, Vogt SL, Deng W, Creus-Cuadros A, Huus KE, Cirstea M, Gerrie M, Barcik W, Yu H, et al. Type VI secretion systems of pathogenic and commensal bacteria mediate niche occupancy in the gut. Cell Rep. 2022;39(4):110731. doi:10.1016/j.celrep.2022.110731. 35476983

[32] Navarro-Garcia F, Ruiz-Perez F, Cataldi A, Larzabal M. Type VI Secretion System in Pathogenic *Escherichia coli*: Structure, Role in Virulence, and Acquisition. Front Microbiol. 2019;10:1965. doi:10.3389/fmicb.2019.01965. 31543869 PMC6730261

[33] Dudley EG, Thomson NR, Parkhill J, Morin NP, Nataro JP. Proteomic and microarray characterization of the AggR regulon identifies a pheU pathogenicity island in enteroaggregative *Escherichia coli*. Mol Microbiol. 2006;61(5):1267–1282. doi:10.1111/j.1365-2958.2006.05281.x. 16925558

[34] Johnson JR, Johnston B, Kuskowski MA, Nougayrede JP, Oswald E. Molecular epidemiology and phylogenetic distribution of the *Escherichia coli* pks genomic island. J Clin Microbiol. 2008;46(12):3906–3911. doi:10.1128/JCM.00949-08. 18945841 PMC2593299

[35] LaCourse KD, Peterson SB, Kulasekara HD, Radey MC, Kim J, Mougous JD. Conditional toxicity and synergy drive diversity among antibacterial effectors. Nat microbiol. 2018;3(4):440–446. doi:10.1038/s41564-018-0113-y. 29459733 PMC5876133

[36] Sahl JW, Steinsland H, Redman JC, Angiuoli SV, Nataro JP, Sommerfelt H, Rasko DA. A comparative genomic analysis of diverse clonal types of enterotoxigenic *Escherichia coli* reveals pathovar-specific conservation. Infect Immun. 2011;79(2):950–960. doi:10.1128/IAI.00932-10. 21078854 PMC3028850

[37] Batut B, Knibbe C, Marais G, Daubin V. Reductive genome evolution at both ends of the bacterial population size spectrum. Nat Rev Microbiol. 2014;12(12):841–850. doi:10.1038/nrmicro3331. 25220308

[38] Imhof M, Schlotterer C. Fitness effects of advantageous mutations in evolving *Escherichia coli* populations. Proc Natl Acad Sci U S A. 2001;98(3):1113–1117. doi:10.1073/pnas.98.3.1113. 11158603 PMC14717

[39] Kibota TT, Lynch M. Estimate of the genomic mutation rate deleterious to overall fitness in *E. coli*. Nature. 1996;381(6584):694–696. doi:10.1038/381694a0. 8649513

[40] Salomon D, Kinch LN, Trudgian DC, Guo X, Klimko JA, Grishin NV, Mirzaei H, Orth K. Marker for type VI secretion system effectors. Proc Natl Acad Sci U S A. 2014;111(25):9271–9276. doi:10.1073/pnas.1406110111. 24927539 PMC4078801

[41] Jana B, Fridman CM, Bosis E, Salomon D. A modular effector with a DNase domain and a marker for T6SS substrates. Nat Commun. 2019;10(1):3595. doi:10.1038/s41467-019-11546-6. 31399579 PMC6688995

[42] Kanarek K, Fridman CM, Bosis E, Salomon D. The RIX domain defines a class of polymorphic T6SS effectors and secreted adaptors. Nat Commun. 2023;14(1):4983. doi:10.1038/s41467-023-40659-2. 37591831 PMC10435454

[43] Gunther P, Quentin D, Ahmad S, Sachar K, Gatsogiannis C, Whitney JC, Raunser S. Structure of a bacterial Rhs effector exported by the type VI secretion system. PLoS Pathog. 2022;18(1):e1010182. doi:10.1371/journal.ppat.1010182. 34986192 PMC8765631

[44] Waneskog M, Halvorsen T, Filek K, Xu F, Hammarlof DL, Hayes CS, Braaten BA, Low DA, Poole SJ, Koskiniemi S. *Escherichia coli* EC93 deploys two plasmid-encoded class I contact-dependent growth inhibition systems for antagonistic bacterial interactions. Microb Genom. 2021;7(3). doi:10.1099/mgen.0.000534. PMC8190604 33646095

[45] Suarez G, Sierra JC, Sha J, Wang S, Erova TE, Fadl AA, Foltz SM, Horneman AJ, Chopra AK. Molecular characterization of a functional type VI secretion system from a clinical isolate of *Aeromonas hydrophila*. Microb Pathog. 2008;44(4):344–361. doi:10.1016/j.micpath.2007.10.005. 18037263 PMC2430056

[46] Marcusson LL, Frimodt-Moller N, Hughes D. Interplay in the selection of fluoroquinolone resistance and bacterial fitness. PLoS Pathog. 2009;5(8):e1000541. doi:10.1371/journal.ppat.1000541. 19662169 PMC2714960

[47] Porter CK, Riddle MS, Tribble DR, Louis Bougeois A, McKenzie R, Isidean SD, Sebeny P, Savarino SJ. A systematic review of experimental infections with enterotoxigenic *Escherichia coli* (ETEC. Vaccine. 2011;29(35):5869–5885. doi:10.1016/j.vaccine.2011.05.021. 21616116

[48] Korajkic A, Wanjugi P, Brooks L, Cao Y, Harwood VJ. Persistence and decay of fecal microbiota in aquatic habitats. Microbiol Mol Biol Rev. 2019;83(4). doi:10.1128/MMBR.00005-19. PMC7405076 31578217

[49] Inglis RF, Bayramoglu B, Gillor O, Ackermann M. The role of bacteriocins as selfish genetic elements. Biol Lett. 2013;9(3):20121173. doi:10.1098/rsbl.2012.1173. 23616642 PMC3645024

[50] Ruhe ZC, Nguyen JY, Chen AJ, Leung NY, Hayes CS, Low DA. CDI systems are stably maintained by a cell-contact mediated surveillance mechanism. PLoS Genet. 2016;12(6):e1006145. doi:10.1371/journal.pgen.1006145. 27355474 PMC4927057

[51] Diez-Gonzalez F. Applications of bacteriocins in livestock. Curr Issues Intest Microbiol. 2007;8:15–23. 17489435

[52] Jeziorowski A, Gordon DM. Evolution of microcin V and colicin Ia plasmids in *Escherichia coli*. J Bacteriol. 2007;189(19):7045–7052. doi:10.1128/JB.00243-07. 17644607 PMC2045219

[53] Waters VL, Crosa JH. Colicin V virulence plasmids. Microbiol Rev. 1991;55(3):437–450. doi:10.1128/mr.55.3.437-450.1991. 1943995 PMC372828

[54] Gerdes K, Rasmussen PB, Molin S. Unique type of plasmid maintenance function: postsegregational killing of plasmid-free cells. Proc Natl Acad Sci U S A. 1986;83(10):3116–3120. doi:10.1073/pnas.83.10.3116. 3517851 PMC323463

[55] Journet L, Cascales E. The type VI secretion system in *Escherichia coli* and related species. EcoSal Plus. 2016;7(1). doi:10.1128/ecosalplus.ESP-0009-2015. PMC11575709 27223818

[56] Nataro JP, Deng Y, Cookson S, Cravioto A, Savarino SJ, Guers LD, Levine MM, Tacket CO. Heterogeneity of enteroaggregative *Escherichia coli* virulence demonstrated in volunteers. J Infect Dis. 1995;171(2):465–468. doi:10.1093/infdis/171.2.465. 7844392

[57] Koskiniemi S, Garza-Sanchez F, Sandegren L, Webb JS, Braaten BA, Poole SJ, Andersson DI, Hayes CS, Low DA. Selection of orphan Rhs toxin expression in evolved *Salmonella enterica* serovar Typhimurium. PLoS Genet. 2014;10(3):e1004255. doi:10.1371/journal.pgen.1004255. 24675981 PMC3967940

[58] Mulder DT, Cooper CA, Coombes BK. Type VI secretion system-associated gene clusters contribute to pathogenesis of *Salmonella enterica* serovar typhimurium. Infect Immun. 2012;80(6):1996–2007. doi:10.1128/IAI.06205-11. 22493086 PMC3370595

[59] Kung VL, Khare S, Stehlik C, Bacon EM, Hughes AJ, Hauser AR. An rhs gene of *Pseudomonas aeruginosa* encodes a virulence protein that activates the inflammasome. Proc Natl Acad Sci USA. 2012;109(4):1275–1280. doi:10.1073/pnas.1109285109. 22232685 PMC3268321

[60] Jones C, Hachani A, Manoli E, Filloux A. An rhs gene linked to the second type VI secretion cluster is a feature of the *Pseudomonas aeruginosa* strain PA14. J Bacteriol. 2014;196(4):800–810. doi:10.1128/JB.00863-13. 24317402 PMC3911176

[61] Steinsland H, Valentiner-Branth P, Perch M, Dias F, Fischer TK, Aaby P, Molbak K, Sommerfelt H. Enterotoxigenic *Escherichia coli* infections and diarrhea in a cohort of young children in Guinea-Bissau. J Infect Dis. 2002;186(12):1740–1747. doi:10.1086/345817. 12447759

[62] Valentiner-Branth P, Steinsland H, Fischer TK, Perch M, Scheutz F, Dias F, Aaby P, Molbak K, Sommerfelt H. Cohort study of Guinean children: incidence, pathogenicity, conferred protection, and attributable risk for enteropathogens during the first 2 years of life. J Clin Microbiol. 2003;41(9):4238–4245. doi:10.1128/JCM.41.9.4238-4245.2003. 12958251 PMC193811

[63] Datsenko KA, Wanner BL. One-step inactivation of chromosomal genes in *Escherichia coli* K-12 using PCR products. Proc Natl Acad Sci U S A. 2000;97(12):6640–6645. doi:10.1073/pnas.120163297. 10829079 PMC18686

[64] Sharan SK, Thomason LC, Kuznetsov SG, Court DL. Recombineering: a homologous recombination-based method of genetic engineering. Nat Protoc. 2009;4(2):206–223. doi:10.1038/nprot.2008.227. 19180090 PMC2790811

[65] Guzman LM, Belin D, Carson MJ, Beckwith J. Tight regulation, modulation, and high-level expression by vectors containing the arabinose PBAD promoter. J Bacteriol. 1995;177(14):4121–4130. doi:10.1128/jb.177.14.4121-4130.1995. 7608087 PMC177145

[66] Kolmogorov M, Bickhart DM, Behsaz B, Gurevich A, Rayko M, Shin SB, Kuhn K, Yuan J, Polevikov E, Smith TPL, et al. metaFlye: scalable long-read metagenome assembly using repeat graphs. Nat Methods. 2020;17(11):1103–1110. doi:10.1038/s41592-020-00971-x. 33020656 PMC10699202

[67] Wick RR, Schultz MB, Zobel J, Holt KE. Bandage: interactive visualization of de novo genome assemblies. Bioinformatics. 2015;31(20):3350–3352. doi:10.1093/bioinformatics/btv383. 26099265 PMC4595904

[68] Wick RR, Holt KE. Polypolish: short-read polishing of long-read bacterial genome assemblies. PLoS Comput Biol. 2022;18(1):e1009802. doi:10.1371/journal.pcbi.1009802. 35073327 PMC8812927

[69] Seemann T. Prokka: rapid prokaryotic genome annotation. Bioinformatics. 2014;30(14):2068–2069. doi:10.1093/bioinformatics/btu153. 24642063

[70] Camacho C, Coulouris G, Avagyan V, Ma N, Papadopoulos J, Bealer K, Madden TL. BLAST+: architecture and applications. BMC Bioinform. 2009;10:421. doi:10.1186/1471-2105-10-421. PMC2803857 20003500

[71] Alanjary M, Steinke K, Ziemert N. AutoMLST: an automated web server for generating multi-locus species trees highlighting natural product potential. Nucleic Acids Res. 2019;47(W1):W276–W282. doi:10.1093/nar/gkz282. 30997504 PMC6602446

[72] Minh BQ, Schmidt HA, Chernomor O, Schrempf D, Woodhams MD, von Haeseler A, Lanfear R. IQ-TREE 2: new models and efficient Methods for phylogenetic inference in the genomic Era. Mol Biol Evol. 2020;37(5):1530–1534. doi:10.1093/molbev/msaa015. 32011700 PMC7182206

[73] Jolley KA, Maiden MC. Bigsdb: scalable analysis of bacterial genome variation at the population level. BMC Bioinform. 2010;11:595. doi:10.1186/1471-2105-11-595. PMC3004885 21143983

[74] Pritchard L, Glover RH, Humphris S, Elphinstone JG, Toth IK. Genomics and taxonomy in diagnostics for food security: soft-rotting enterobacterial plant pathogens. Anal Methods-Uk. 2016;8(1):12–24. doi:10.1039/C5AY02550H.

[75] Zhang J, Guan J, Wang M, Li G, Djordjevic M, Tai C, Wang H, Deng Z, Chen Z, Ou HY. SecReT6 update: a comprehensive resource of bacterial type VI secretion systems. Sci China Life Sci. 2023;66(3):626–634. doi:10.1007/s11427-022-2172-x. 36346548

[76] Ranwez V, Harispe S, Delsuc F, Douzery EJ. MACSE: multiple alignment of coding SEquences accounting for frameshifts and stop codons. PLoS ONE. 2011;6(9):e22594. doi:10.1371/journal.pone.0022594. 21949676 PMC3174933

[77] Bromberg Y, Rost B. SNAP: predict effect of non-synonymous polymorphisms on function. Nucleic Acids Res. 2007;35(11):3823–3835. doi:10.1093/nar/gkm238. 17526529 PMC1920242

[78] Murrell B, Moola S, Mabona A, Weighill T, Sheward D, Kosakovsky Pond SL, Scheffler K. FUBAR: a fast, unconstrained bayesian approximation for inferring selection. Mol Biol Evol. 2013;30(5):1196–1205. doi:10.1093/molbev/mst030. 23420840 PMC3670733

[79] Eddy SR. A new generation of homology search tools based on probabilistic inference. Genome Inform. 2009;23:205–211. 20180275

[80] Hauser M, Steinegger M, Soding J. Mmseqs software suite for fast and deep clustering and searching of large protein sequence sets. Bioinformatics. 2016;32(9):1323–1330. doi:10.1093/bioinformatics/btw006. 26743509

[81] Yoon SH, Ha SM, Lim J, Kwon S, Chun J. A large-scale evaluation of algorithms to calculate average nucleotide identity. Antonie Van Leeuwenhoek. 2017;110(10):1281–1286. doi:10.1007/s10482-017-0844-4. 28204908

[82] Zulkower V, Rosser S. DNA features viewer: a sequence annotation formatting and plotting library for Python. Bioinformatics. 2020;36(15):4350–4352. doi:10.1093/bioinformatics/btaa213. 32637988

