# Supplementary material for: Colicins and T6SS-based competition systems enhance enterotoxigenic E. coli (ETEC) competitiveness
Source: Gut Microbes. 2023 Dec 27;16(1):2295891. doi: 10.1080/19490976.2023.2295891 (PMC10761095; doi:10.1080/19490976.2023.2295891)
Supplement: Supplemental_material_revised 231201 clean.docx [file KGMI_A_2295891_SM3393.docx]

**Supplemental material for:**

**Colicins and T6SS-based competition systems enhance Enterotoxigenic *E. coli* (ETEC) competitiveness**

Jonas Kjellin^1†^, Danna Lee^1†^, Hans Steinsland^3,4^, Rachel Dwane^1^, Oda Barth Vedoy^2^, Kurt Hanevik^2,5^, Sanna Koskiniemi^1*^

**This supplement contains:**

**Figure S1 legend p. 2**

**Figure S2 p. 3**

**Figure S3 p. 4**

**Table S1 p. 5**

**Table S2 legend p. 6**

**Table S3 p. 7**

**Table S4 p. 8**

**Figure S4 p. 9**

**Table S5 legend p. 9**

**Table S6 legend p. 9**

**Figure S5 p. 10**

**Table S7 legend p. 11**

**Table S8 p. 11**

**Table S9 p. 12**

**Table S10 p. 13**

**Table S11 legend p. 13**

**Table S12 legend p. 13**

**References p. 14**

**Figure S1. Phylogeny and competition systems in 94 ETEC strains and 11 non-ETEC *E. coli* and *Shigella* strains using *E. fergusonii* as outgroup.** Non-ETEC strains are highlighted in red. Phylogeny is based on an alignment of 100 single copy genes and analyzed with IQ-TREE (for more detail see Methods) [1] based on the maximum likelihood method and the Generalised time reversible (GTR) substitution model with 1000 bootstrap replicates. The bootstrap value for each branch is shown in red. The presence and types of bacteriocins, contact dependent inhibition (CDI) systems and type 6 secretion systems (T6SS) are indicated in green. T6SS systems with one or more core gene appear to be non-functional or missing is indicated in yellow. T6SS effectors (Hcp or PAAR-Rhs) are defined by their respective protein domain and indicated with green if present. Effectors were only analyzed for ETEC strains with predicted functional T6SS (strains lacking a predicted functional T6SS are indicated in grey). T6SS effectors with no identifiable C-terminal domain were clustered based on sequence similarity (Ct-1 to Ct-17). Also, each Rhs effector were group based on genomic location where the same number indicates that the effectors are located at similar location in respective genome. **Figure S1 is found in a separate file.**


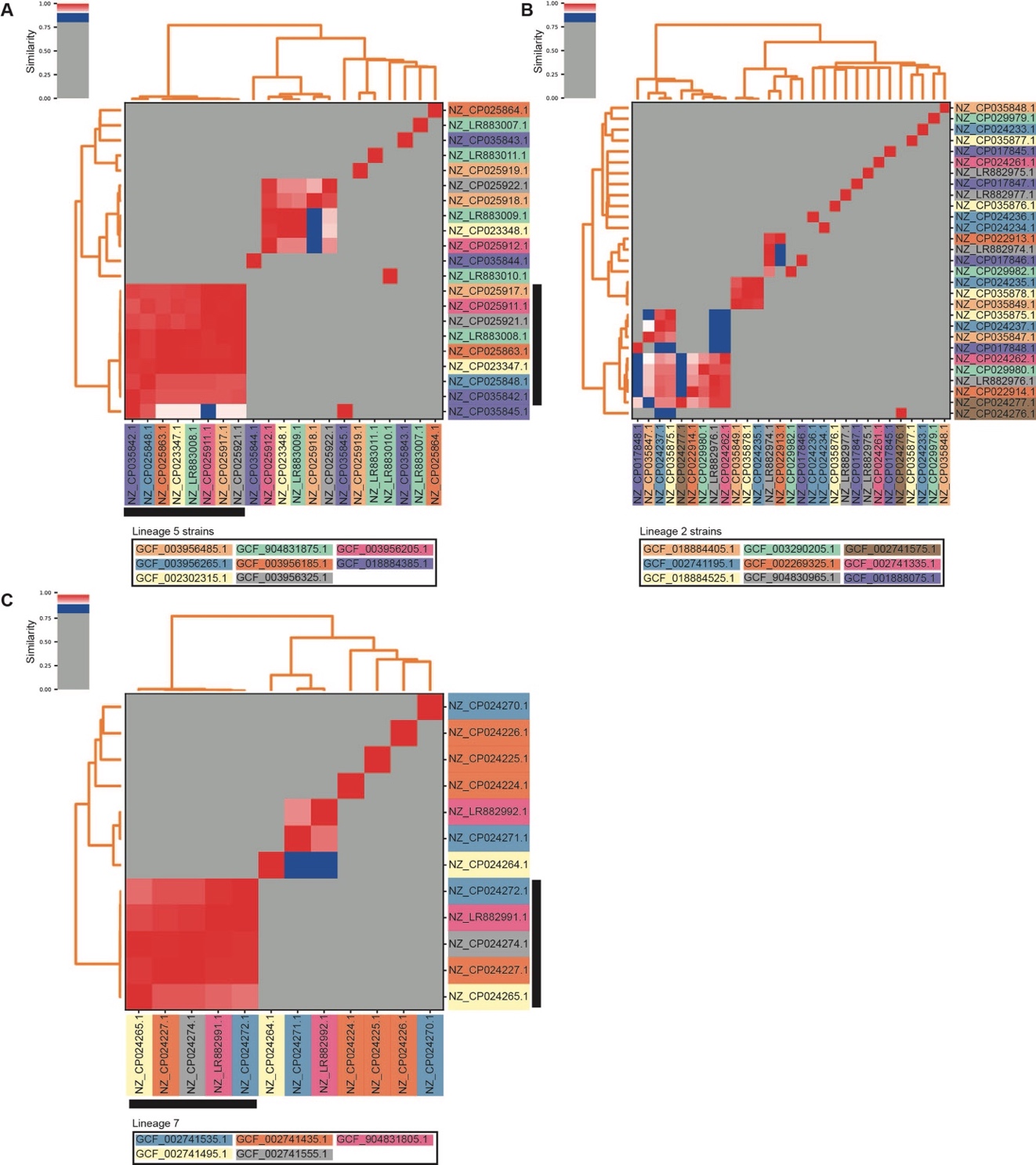


**Figure S2. Comparison of plasmids carried by strains within lineage 2, 5 and 7.** Sequence comparison of different plasmids found in **A)** Lineage 5, **B)** Lineage 2 and **C)** in Lineage 7 performed with pyani v. 0.2.12. Similarity between two sequences is calculated as the proportion of nucleotide identity multiplied with the proportion of alignment coverage. Thus, the maximum similarity of 1.0 represents plasmid sequences that are 100% identical and completely overlaps with each other. GenBank plasmid accession numbers are given on the axes and are colored based on which strain they belong to. Strain colors are specified in the legend of each subfigure. Black solid lines next to the plasmid accession numbers indicates plasmids that are present in all members of respective lineage, i.e. Colicin Ia plasmid in A and STp, CS6 encoding plasmid in C.

**Figure S3. Sequence similarity and synteny mapping of Colicin Ia encoding ETEC plasmids**. **A)** Whole plasmid comparison of Colicin Ia encoding plasmids from all clades were the *col-Ia* gene was identified. Black solid line indicates length of plasmid in kilobases and syntenic blocks (≥ 80% similar) are connected with dashed grey lines. Blocks above the solid black line (turquoise) and below (green) indicate if the syntenic region is located on the forward or reverse strand, respectively. Since the Lineage 5 plasmids were close to identical, plasmid from only one representative strain is shown. Plasmids are named after the strain's accession number and colored based on which lineage/clade they belong to. **B)** The *col-Ia* locus for each plasmid. Homologous genes are indicated with dashed grey lines. Loci of TW10722 and GCF_002741455.1 have been reorientated so that *col-Ia* gene is on the same strand in each strain to facilitate comparison.

**Table S1.** **Whole genome sequencing summary and assembly statistics for TW10690, TW10573, TW14425 and TW10828.** All genomes were assembled from Nanopore long-read sequences. Short-read polishing was performed of the assemblies for TW10690 and TW10573 due to the low coverage for some of the plasmids in the long-read sequencing.

| **Strain** | **Nanopore** | | | | **Illumina Polypolish** | | | **Pre-polish accuracy** |
| --- | --- | --- | --- | --- | --- | --- | --- | --- |
|  | **Contig** | **GenBank accession** | **Length (kb)** | **Depth** | **Depth** | **Coverage** | **Corrections** |  |
| TW10690 | Chromosome | CP127034 | 4853,4 | 336 | 526,3 | 100,00% | 81 | 100,00% |
| TW10690 | pTW10690_129.5k | CP127033 | 129,5 | 179 | 972,8 | 100,00% | 37 | 99,97% |
| TW10690 | pTW10690_70.6k | CP127031 | 70,6 | 75 | 763,8 | 100,00% | 18 | 99,98% |
| TW10690 | pTW10690_4.2k | CP127032 | 4,2 | 29 | 3812 | 99,95% | 18 | 99,95% |
| TW10573 | Chromosome | CP127035 | 5015,1 | 336 | 493,3 | 100,00% | 111 | 99,98% |
| TW10573 | pTW10573_90.4k | CP127038 | 90,4 | 38 | 681,9 | 100,00% | 35 | 99,96% |
| TW10573 | pTW10573_78.2k | CP127037 | 78,2 | 224 | 768,6 | 100,00% | 71 | 99,91% |
| TW10573 | pTW10573_9.3k | CP127036 | 9,3 | 27 | 5615 | 99,98% | 1 | 99,99% |
| TW14425 | Chromosome | CP127265 | 4943,6 | 742 | N/A | N/A | N/A | N/A |
| TW14425 | pTW14425_5.9k | CP127267 | 5,9 | 151 | N/A | N/A | N/A | N/A |
| TW14425 | pTW14425_76.4k | CP127266 | 76,4 | 334 | N/A | N/A | N/A | N/A |
| TW14425 | pTW14425_123.7k | CP127268 | 123,7 | 1027 | N/A | N/A | N/A | N/A |
| TW10828 | Chromosome | CP127262 | 4951,8 | 372 | N/A | N/A | N/A | N/A |
| TW10828 | pTW10828_115.1k | CP127264 | 115,1 | 778 | N/A | N/A | N/A | N/A |
| TW10828 | pTW10828_114.6k | CP127263 | 114,6 | 579 | N/A | N/A | N/A | N/A |
| TW10828 | pTW10828_17.3k | CP127261 | 17,3 | 100 | N/A | N/A | N/A | N/A |

**Table S2. Summary of all analyses for each strain included in the study.** Strain order corresponds to the phylogeny in Figure S1. PubMLST-based sequence type (ST), enterotoxins and colonization factors are given for each strain. ETEC lineage assignment according to Mentzer *et al.* [2] is based on sequence type and similarities in encoded colonization factors for each strain. The identified type 6 secretion systems (T6SS status, and Type columns) are considered to be intact if all core components are present. If a component is found but divided into several genes, by e.g. transposon insertion or premature stop codons, it's indicated as split. Truncated components are defined as identified core components with less than 90 % alignment coverage with the reference sequences. T6SS status is a summary of the identified systems and identified as intact if all core components of at least one system is present and complemented if a core component is broken or missing but another version is present at another locus. All other identified T6SS that do not fulfill these criteria are defined as residual. T6SS effectors (Hcp or PAAR-Rhs) are defined by their respective protein domain and indicated with + if present. Only ETEC strains with predicted functional T6SS were analysed. Non-ETEC strains or ETEC strains lacking a predicted functional T6SS are indicated in grey. Effectors with no identifiable domain were grouped based on sequence similarity (Ct-1 to Ct-17). Also, each Rhs effector were group based on genomic location where the same number indicates that the effectors are located at similar location in respective genome. Table S2 is found in a separate file.

**Table S3. Collection year and country for Lineage 5 strains.** Metadata collected from NCBI RefSeq database.

| **Accession** | **Strain** | **Collection year** | **Country** |
| --- | --- | --- | --- |
| GCF_002302315.1 | ETEC-2265 | 2006 | Bangladesh |
| GCF_003956325.1 | 103605 | 2010 | Gambia |
| GCF_003956205.1 | 204446 | 2010 | Mali |
| GCF_904831875.1 | E1779 | 2005 | Bangladesh |
| GCF_003956485.1 | 120899 | 2012 | Gambia |
| GCF_003956185.1 | 504237 | 2010 | India |
| GCF_018884385.1 | TW10722 | 1997 | Guinea-Bissau |
| GCF_003956365.1 | 602354 | 2009 | Bangladesh |

**Table S4. Sequence comparison of Colicin Ia coding sequences from all clades where it was identified.** All *col-Ia* sequences within each clade were identical.

| **Codon position** | **Lineage 5** | | **2 member clade (GCF_018884285.1 & GCF_002741455.1)** | | **1 member clade (GCF_024584865.1)** | |  |
| --- | --- | --- | --- | --- | --- | --- | --- |
|  |  |  |  |  |  |  |  |
| **Site** | **Codon** | **Amino acid** | **Codon** | **Amino acid** | **Codon** | **Amino acid** |  |
| 231, 234 | ACA | T | ACA | T | GCA | A |  |
| 243, 246 | AAC | N | ATG | M | AAC | N |  |
| 267, 270 | GAA | E | GAG | E | GAA | E |  |
| 282, 285 | CGC | R | CGT | R | CGC | R |  |
| 294, 297 | GGA | G | GCA | A | GGA | G |  |
| 300, 303 | AGC | S | CGC | R | CGC | R |  |
| 459, 462 | GAC | D | GAT | D | GAC | D |  |
| 492, 495 | CTG | L | CTC | L | CTC | L |  |
| 1008, 1011 | ACC | T | ACC | T | AGC | S |  |
| 1023, 1026 | GAT | D | GAC | D | GAC | D |  |
| 1134, 1137 | CGA | R | AGA | R | CGA | R |  |
| 1236, 1239 | GAC | D | GAT | D | GAC | D |  |
| 1617, 1620 | GGA | G | GGA | G | GGT | G |  |
| 1656, 1659 | GCT | A | ACT | T | ACT | T |  |
| 1716, 1719 | GCC | A | ACC | T | GCC | A |  |
| 1719, 1722 | ATT | I | ATC | I | ATT | I |  |
| 1749, 1752 | GTT | V | GTG | V | GTT | V |  |

**Figure S4. Representative T6SS loci of each type identified in the study. A)** The strain from which the representative T6SS is derived from is indicated for each type of system, including the accession and coordinates of the genomic position. **B)** Genomic overview of where T6SS and effector loci are found.

**Table S5.** **Raw data for SNAP analysis.** Table S5 is found as a separate file.

**Table S6.** **Raw data for FUBAR analysis.** Table S6 is found as a separate file.

**Figure S5. Representative locus of each type of effector identified in this study.** For each effector, a representative gene order of the 10 kb up-/down-stream is plotted. The representative strain is indicated for each locus together with which different types of effectors is found at that genomic position and number of strains. Each locus is oriented so that the effector is on the forward strand.

**Table S7. Raw data for bacterial growth assays and competitions found in figures 3 and 4.** Table S7 is found as a separate file.

**Table S8. Strains used in the experimental work of the study.**

| **Strain** | **Genotype** | **Origin** |
| --- | --- | --- |
| SK620 | *Eco* K12 MG1655 *lacA-cat* | [3] |
| SK2827 | *Eco* MG1655 *lacA-kanR* | [4] |
| SK3448 | *Eco* ETEC TW10598 | [5] |
| SK3449 | *Eco* ETEC TW10722 | [5] |
| SK3450 | *Eco* ETEC TW11681 | [5] |
| SK5506 | *Eco* ETEC TW10722 *col-Ia::cat* | This work |
| SK5507 | *Eco* ETEC TW10722 *tssM::kan* | This work |
| SK5698 | *Eco* MG1655 *gyrA1*(S83L), *gyrA2*(D87N), *parC*(S80I) | [6] |
| SK6121 | *Eco* ETEC TW14425 | [5] |
| SK6122 | *Eco* ETEC TW10828 | [5] |
| SK6123 | *Eco* ETEC TW10573 | [5] |
| SK6128 | *Eco* ETEC TW10590 | [5] |
| SK6129 | *Eco* ETEC TW10690 | [5] |
| SK6133 | *Eco* ETEC H10407 | [5] |
| SK6189 | *Eco* ETEC TW10722 *tssM::kan, col-Ia::cat* | This work |
| SK6193 | *Eco* ETEC TW10828 *tssM::kan* /pBAD33::*tssM* | This work |
| SK6649 | *Eco* ETEC TW10828 *tssM::kan* | This work |

**Table S9. Oligos used in the experimental work of the study.**

| **Oligo** | **Description** | **Sequence** |
| --- | --- | --- |
| SK387 | pBAD33 screening-F | CGCTTCAGCCATACTTTTCA |
| SK388 | pBAD33 screening-R | GTCTCATGAGCGGATACATATTTG |
| SK841 | pCH450 screening-F | CACGGCGTCACACTTTGC |
| SK842 | pCH450 screening-R | CTCCTGCCACATGAAGCAC |
| SK2037 | TW10722 colIa KO - F | TGTTTTCCTCAGAGGATGAAGGAGATACCGTGTAGGCTGGAGCTGCTTC |
| SK2038 | TW10722 colIa KO - R | TGTAAGATAGGCAGCCTGTCTTATATTTTACATATGAATATCCTCCTTA |
| SK2039 | TW10722 colIa seq - F | CTGCCACAGCGTCAGCAG |
| SK2040 | TW10722 colIa seq - R | CCCGGCCTGATTTCTGGAAC |
| SK2041 | TW10722 tssM KO - F | GGCAGAACAATAAAAGAGAACAGGAGTTTTTGTAGGCTGGAGCTGCTTC |
| SK2042 | TW10722 tssM KO - R | TGTCAGTCCGTTACTATCCATCCCTGCCTCCATATGAATATCCTCCTTA |
| SK2043 | TW10722 tssM seq - F | GGCTGGTACAGCTTGCGC |
| SK2044 | TW10722 tssM seq - R | AGTGCCAGCGACTCCACC |
| SK2053 | TW10722 colIa I - Smal - F | ATATcccgggGTGCAAAAGAAAGGAAAACAGGCAG |
| SK2054 | TW10722 colIa I - SalI - R | GCGCgtcgacGCTGCCTATCTTACAGACAGCC |
| SK2529 | TW10828 tssM KO - F | CGGCAGAACAATAAAAGAGAACAGGAGTTTTTGTGTTCA  GTGTAGGCTGGAGCTGCTTC |
| SK2531 | TW10828 tssM KO - R | TGTCAGTCCGTTACTATCCATCCCTGCCTCCTCAGTACAGGGTC  CATATGAATATCCTCCTTA |
| SK2737 | TW10828 tssM-XbaI-F | ATATtctagaGAGAACAGGAGTTTTTGTGTTC |
| SK2738 | TW10828 tssM-HindIII-R | GCGCaagcttCTCAGTACAGTGTGTCTGAC |

**Table S10. Accession numbers for reference sequences used to identify ETEC enterotoxins and colonization factors.**

| **Enterotoxin/Colonization factor** | **Uniprot accession** | **NCBI accession** |
| --- | --- | --- |
| Heat-labile enterotoxin A chain | sp\|P06717\|ELAP_ECOLX |  |
| Heat-labile enterotoxin B chain | sp\|P0CK94\|ELBH_ECOLX |  |
| Heat-labile enterotoxin B chain | sp\|P32890\|ELBP_ECOLX |  |
| Heat-labile enterotoxin IIB, A chain | sp\|P43528\|E2BA_ECOLX |  |
| Heat-labile enterotoxin IIA, A chain | sp\|P13810\|E2AA_ECOLX |  |
| Heat-labile enterotoxin IIB, B chain | sp\|P43529\|E2BB_ECOLX |  |
| Heat-labile enterotoxin A chain | sp\|P43530\|ELAH_ECOH1 |  |
| Heat-labile enterotoxin B chain | sp\|D0Z6T1\|ELBH_ECOH1 |  |
| Heat-labile enterotoxin IIA, B chain | sp\|P13812\|E2AB_ECOLX |  |
| Heat-stable enterotoxin A3/A4 | sp\|P07965\|HST3_ECOLX |  |
| Heat-stable enterotoxin ST-IA/ST-P | sp\|P01559\|HST1_ECOLX |  |
| Heat-stable enterotoxin II | sp\|P22542\|HSTI_ECOLX |  |
| Heat-stable enterotoxin ST-2 | sp\|P01560\|HSTB_ECOLX |  |
| Heat-stable enterotoxin A2 | sp\|Q47185\|HST2_ECOLX |  |
| CS1 |  | WP_000768757.1 |
| CS2 |  | WP_000773452.1 |
| CS3 |  | WP_013188494.1 |
| CS4 |  | WP_024170052.1 |
| CS5 |  | WP_000739895.1 |
| CS6 |  | WP_000750952.1 |
| CS7 |  | WP_099550625.1 |
| CS8 |  | WP_110409206.1 |
| CS12 |  | WP_088568720.1 |
| CS13 |  | WP_074512221.1 |
| CS14 |  | WP_000768758.1 |
| CS15 |  | WP_164158208.1 |
| CS17 |  | CAA66126.1 |
| CS18 |  | WP_265745017.1 |
| CS19 |  | WP_074502572.1 |
| CS20 |  | AAL31637.1 |
| CS21 |  | KPQ45498.1 |
| CS21 |  | CAD6023170.1 |
| CS22 |  | WP_164720171.1 |
| CFA/I |  | WP_000669509.1 |
| K88 |  | WP_000751751.1 |
| 987P |  | WP_114140022.1 |
| F18 |  | WP_122986601.1 |

**Table S11. Similarity control of assemblies with the same sequence type and similar repertoire of competition system.** Similarity was calculated by multiplying average nucleotide identity and alignment coverage. Table S11 is found in a separate file.

**Table S12. Accession number and sequence of all bacteriocins and T6SS effectors identified in this study.** Table S12 is found in a separate file.

**References**

1. Minh, B.Q., H.A. Schmidt, O. Chernomor, D. Schrempf, M.D. Woodhams, A. von Haeseler, and R. Lanfear, *IQ-TREE 2: New Models and Efficient Methods for Phylogenetic Inference in the Genomic Era.* Mol Biol Evol, 2020. **37**(5): p. 1530-1534.

2. von Mentzer, A., T.R. Connor, L.H. Wieler, T. Semmler, A. Iguchi, N.R. Thomson, D.A. Rasko, E. Joffre, J. Corander, D. Pickard, G. Wiklund, A.M. Svennerholm, A. Sjoling, and G. Dougan, *Identification of enterotoxigenic Escherichia coli (ETEC) clades with long-term global distribution.* Nat Genet, 2014. **46**(12): p. 1321-6.

3. Ghosh, A., O. Baltekin, M. Waneskog, D. Elkhalifa, D.L. Hammarlof, J. Elf, and S. Koskiniemi, *Contact-dependent growth inhibition induces high levels of antibiotic-tolerant persister cells in clonal bacterial populations.* EMBO J, 2018. **37**(9).

4. Virtanen, P., M. Waneskog, and S. Koskiniemi, *Class II contact-dependent growth inhibition (CDI) systems allow for broad-range cross-species toxin delivery within the Enterobacteriaceae family.* Mol Microbiol, 2019. **111**(4): p. 1109-1125.

5. Steinsland, H., D.W. Lacher, H. Sommerfelt, and T.S. Whittam, *Ancestral lineages of human enterotoxigenic Escherichia coli.* J Clin Microbiol, 2010. **48**(8): p. 2916-24.

6. Marcusson, L.L., N. Frimodt-Moller, and D. Hughes, *Interplay in the selection of fluoroquinolone resistance and bacterial fitness.* PLoS Pathog, 2009. **5**(8): p. e1000541.
